# *w*Mel replacement of dengue-competent mosquitoes is robust to near-term climate change

**DOI:** 10.1038/s41558-023-01746-w

**Published:** 2023-08-03

**Authors:** Váleri N. Vásquez, Lara M. Kueppers, Gordana Rašić, John M. Marshall

**Affiliations:** 1 Energy and Resources Group, University of California, Berkeley, CA USA; 2 Department of Electrical Engineering and Computer Sciences, College of Engineering, University of California, Berkeley, CA USA; 3 Climate and Ecosystem Sciences Division, Lawrence Berkeley National Laboratory, Berkeley, CA USA; 4 Mosquito Genomics, QIMR Berghofer Medical Research Institute, Brisbane, Queensland Australia; 5 Divisions of Epidemiology and Biostatistics, School of Public Health, University of California, Berkeley, CA USA; 6 Innovative Genomics Institute, University of California, Berkeley, CA USA

**Keywords:** Ecological epidemiology, Ecological modelling

## Abstract

Rising temperatures are impacting the range and prevalence of mosquito-borne diseases. A promising biocontrol technology replaces wild mosquitoes with those carrying the virus-blocking *Wolbachia* bacterium. Because the most widely used strain, *w*Mel, is adversely affected by heat stress, we examined how global warming may influence *w*Mel-based replacement. We simulated interventions in two locations with successful field trials using Coupled Model Intercomparison Project Phase 5 climate projections and historical temperature records, integrating empirical data on *w*Mel’s thermal sensitivity into a model of *Aedes aegypti* population dynamics to evaluate introgression and persistence over one year. We show that in Cairns, Australia, climatic futures necessitate operational adaptations for heatwaves exceeding two weeks. In Nha Trang, Vietnam, projected heatwaves of three weeks and longer eliminate *w*Mel under the most stringent assumptions of that symbiont’s thermal limits. We conclude that this technology is generally robust to near-term (2030s) climate change. Accelerated warming may challenge this in the 2050s and beyond.

## Abstract (web summary)

The biocontrol technology (*w*Mel) used to mitigate mosquito-borne viruses is adversely affected by heat stress. The authors integrate empirical data on mosquito population dynamics and *w*Mel thermal sensitivity to show that the technology is generally robust to near-term climate change.

## Main

Temperature influences both mosquito and pathogen traits, affecting the geographical range and prevalence of mosquito-borne diseases like malaria, dengue and Zika virus^1–5^. Anthropogenic warming is expected to have heterogenous impacts, with increases in seasonal suitability for the malarial *Anopheles* mosquito in some regions and extensions of the environmental conditions favourable to arbovirus vectors, such as the *Aedes* mosquito, in others^6^.

Disease risk under future climates underscores a need for improved vector control tools, particularly as insecticide efficacy wanes due to evolved resistance^7–9^. There has been a surge of innovation in control technologies over the past decade, including several successful field trials of a self-sustaining biocontrol approach called *Wolbachia*-based replacement^10–12^. This technique, which transfects wild mosquitoes with a maternally inherited endosymbiotic bacterium that is naturally occurring in many arthropods, can be highly effective at blocking the ability to transmit human pathogens^13^. Multiple strains of the *Wolbachia* bacterium, each bearing unique biological properties, have been transferred into various *Aedes* species^14^. Public health intervention trials of *Aedes aegypti* transfected with the *w*Mel strain^11^ have been undertaken in Latin America, Asia and Oceania^15–18^. Most *Wolbachia* replacement efforts trialled for control of *Aedes*-borne viruses have used the *w*Mel strain naturally occurring in *Drosophila melanogaster*^19^.

In transfected *A. aegypti* mosquitoes, *w*Mel reliably causes stable maternal transmission and blocks replication of pathogenic viruses while incurring a relatively low fitness cost compared to alternative *Wolbachia* strains^20^. Under optimal laboratory conditions, *w*Mel demonstrates complete cytoplasmic incompatibility (CI), which renders crosses between infected males and uninfected females unviable, favouring the reproduction of *w*Mel carriers and driving its own spread through a vector population. However, recent studies have shown that CI—a property vital to *w*Mel utility—is weakened by the cyclical heat stress that can characterize field conditions^21^. High temperatures also decrease the hatch rate of *w*Mel-infected eggs and reduce *w*Mel density in adults, impairing maternal transmission and causing the infection to fall out of a laboratory population once a threshold of 35.0 °C is reached^22^. Another strain, *w*AlbB, exhibits less susceptibility to high rearing temperatures and has been successfully established in Malaysian populations of *A. aegypti*^23^; however, its predominant use has been for the suppression (population lowering) of that vector^24–26^.

Using projections of future increases in the frequency, severity and duration of heatwaves^27^, together with laboratory evidence of the negative impact that oscillating temperature regimes and thermal shocks have on *w*Mel infection^21,22^, we tested our hypothesis that the hotter, more prevalent heat extremes and higher average temperatures brought by climate change could impede *w*Mel-based replacement interventions. We assess the effects of alternative climate scenarios on this technology in Cairns, Queensland, Australia—the location of the first successful field trial^10^—using entomological end points, including the frequency of *w*Mel infection and the magnitude of wild-type (WT) suppression, to examine introgression and fixation in a simulated *A. aegypti* population over a year-long horizon. We investigate the limits of our findings through analyses of *w*Mel interventions in the region of Nha Trang City, Vietnam, where the technology has also been trialled and where higher temperatures and longer heatwaves are expected. Our computational experiments study the compounding interaction of warming trends, *w*Mel*’s* intrinsic biological thresholds and extrinsic fitness costs, and uncertainties about the functional form of temperature impacts on *w*Mel to understand the resilience of the technology to climate change via the dynamics of its introduction and short-term persistence.

## Results

**Fig. 1:**
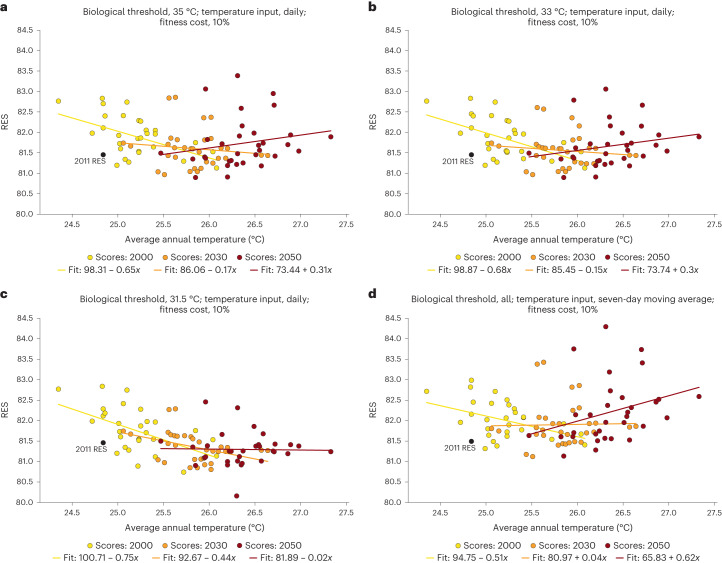
Impact of average temperature change on the Replacement Efficacy Score of *w*Mel. Increases in average temperature impact the RES of *Wolbachia*-based interventions. Each circle marker represents an RES for the release simulated in an individual year, with colours corresponding to the temperature regime (2011 in black, all other historical in yellow, 2030 in orange, 2050 in red). The trend line fits are noted in each panel legend. **a**–**c**, Results from the sensitivity analysis for unique thermal thresholds (35 °C (**a**) 33 °C (**b**), 31.5 °C (**c**)) and daily temperature inputs to the *w*Mel dynamic model. **d**, Results when seven-day moving averages are used as temperature inputs; all three thermal thresholds returned equivalent output under this assumption.

*w*Mel-based population replacement is robust to projected regional warming trends out to mid-century. Outcomes were quantified using the replacement efficacy score (RES), a metric modelled after the suppression efficacy score (SES)^28^, and evaluated on a 0–100 scale relative to ideal efficacy (sustained fixation). The RES summarizes the success with which the standing population is substituted by *w*Mel carriers. It serves as a basis of comparison to assess the replacement achieved by alternative scenarios (Methods). Across the scenarios, RES scores remained above 79 (Fig. 1 and section 5 in the Supplementary Information).

We examined the consequences of CI and maternal inheritance—both thermally sensitive biological mechanisms—failing completely when daily average temperature reached the 35.0 °C threshold, which has been empirically shown to drop the *w*Mel titre to zero in exposed *A. aegypti* eggs. We also conducted a sensitivity analysis where CI and maternal inheritance fail at lower temperatures (33.0 °C and 31.5 °C). For interventions conducted under Cairns average daily temperatures in both historical (1990–2019) and projected future (2024–2039, 2044–2059) years, RES scores and their correlation with temperature were nearly identical between the 35.0 °C and 33.0 °C threshold scenarios (Fig. 1a,b). The lowest threshold, 31.5 °C, produced outcomes that were substantially more sensitive to temperature than those generated by the 35.0 °C and 33.0 °C alternatives (Fig. 1c).

Because the data used to develop *w*Mel temperature-responsive dynamics were from laboratory studies conducted at a resolution of one week, we performed a sensitivity analysis to investigate the effect of altering the functional form of temperature inputs. In one set of simulations, temperature inputs to the mechanistic model assumed that the same impact observed after one week of exposure would also occur after one day. In a second set of simulations, a seven-day moving average constituted the temperature inputs. The simulations driven by moving averages resulted in equivalent output under all threshold values (35.0 °C, 33.0 °C, 31.5 °C) for CI and maternal inheritance (Fig. 1c,d). These runs also generated a larger difference between the 2030 and 2050 RES scores and trends, compared to results driven by daily temperature inputs. A third sensitivity analysis examined how the *w*Mel-induced fitness cost affected outcomes. The influence was minimal; therefore, all main text results assume a 10% cost on *w*Mel carriers relative to WT *A. aegypti* (section 5 of the SupplementaryInformation for 0% and 20%).

The linear relationship of *w*Mel replacement to increasing temperature diminishes in future, warmer decades. RES scores were calculated for interventions under average daily temperatures across both historical and future years, with the latter encompassing both the Representative Concentration Pathway (RCP) 4.5 and 8.5 scenarios (Fig. 1). Under historical climates, assuming daily inputs to the *w*Mel model and using the thermal threshold of 35 °C, temperature as a linear covariate accounts for approximately 25% (*R*^2^ = 0.2540, *P* < 0.0045) of the variation in the model fit (*y* = 73.44 − 0.31*x*); each degree of temperature increase lowers the RES score by 0.31.

However, the relationship between temperature and RES is non-linear: holding the thermal threshold and daily input assumptions constant during model runs representative of 2024–2039, the effect of annual average temperature on the RES—assuming the highest threshold of 35 °C—is no longer significantly negative (*R*^2^ = 0.0157, *P* < 0.4948). This is also the case in the years 2044–2059 (*R*^2^ = 0.0389, *P* < 0.2788). Given that temperature is the only input variable permitted to change in the model over time, the non-linearity of its association with RES is evidenced by this shift in significance under future scenarios. The changing linear relationships between temperature and RES in future decades reflect the non-linear dynamics in this model system.

Altering the functional form of temperature inputs such that the *w*Mel model is driven by a seven-day moving average yields the same reduction in temperature sensitivity. There is again a negative relationship between temperature and RES score during the historical period (*y* = 94.75 − 0.51*x*); however, annual temperature only accounts for 17.2% (*R*^2^ = 0.17219, *P* < 0.02259) of the variation in that linear fit. In the linear model fit for future decades, the correlation is absent (2030s: *R*^2^ = 0.00048, *P* < 0.90467, 2050s: *R*^2^ = 0.3110, *P* < 0.08316). The primary distinction from the model with daily temperature inputs is that the absolute magnitude of the slope in the linear model fit is invariably larger. This is sensible, given that the moving average approach extends the effect of a single hot day for an additional six days.

**Fig. 2:**
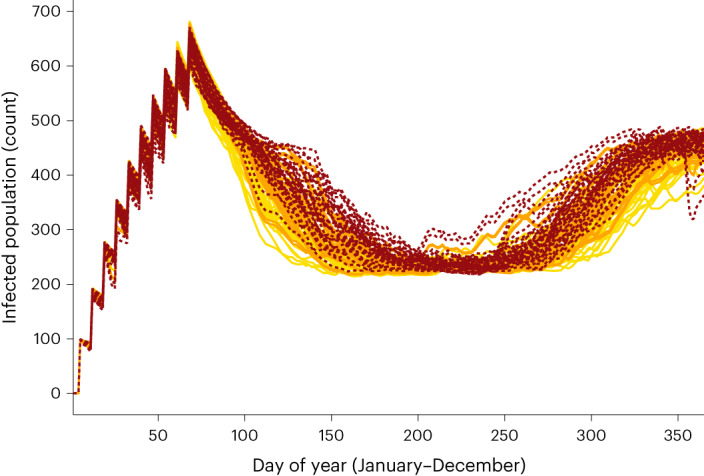
The effect of average temperature on the population dynamics of *w*Mel-infected females lends insight to Replacement Efficacy Score results. Year-on-year population dynamics of *w*Mel-infected females. Each line represents one simulated intervention year; each colour corresponds to a different temperature regime (historical in yellow, 2030 in orange, 2050 in red).

Because the RES is a single value that summarizes success over the entire period of interest, understanding the reason for this metric’s shifting relationship with temperature requires a closer look at the population dynamics (Fig. 2). Under the 2050 scenarios, the number of *w*Mel*-*infected females drops towards the end of the calendar year (summer in the Southern Hemisphere). Thresholds for temperature-sensitive *w*Mel mechanisms (CI and maternal inheritance) are exceeded, leading to the reduction of the infected population. However, higher average temperatures during the coolest part of the year also improve *w*Mel replacement during that period. These mid-year dynamics are strong enough to mask the decrease in infected females that occurs in later months when using a summary metric.

*w*Mel-based population replacement also remains robust to existing predictions of future heatwaves in Cairns for both the 2030 and 2050 climate regimes. For a description of the methodology used to construct the heatwave timeseries, see Methods and section 2 of the Supplementary Information. As with the average temperature change experiments, we conducted sensitivity analyses over the range of biological thermal thresholds, functional form of temperature inputs and fitness costs.

**Fig. 3:**
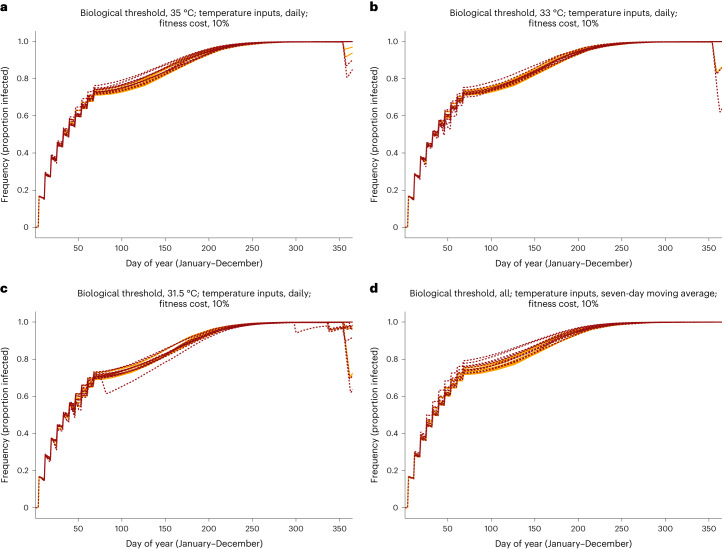
Frequency of wMel infection during future heatwave years. Effect of future heatwaves on the frequency of *w*Mel infection in an adult female *A. aegypti* population. Each line corresponds to one simulated intervention year; each colour corresponds to a different temperature regime (2030 in orange, 2050 in red). Each panel reflects infection frequency given unique thermal thresholds (35 °C (**a**) 33 °C (**b**), 31.5 °C (**c**)) and daily temperature inputs to the *w*Mel dynamic model. **d,** Infection frequency when seven-day moving averages are used as temperature inputs; all three thermal thresholds returned equivalent output under this assumption.

Simulations using daily temperature inputs lower *w*Mel frequency across all thermal thresholds (35.0 °C, 33.0 °C, 31.5 °C) for select years (Fig. 3). This holds for all assumptions of fitness cost. In the most severe cases, the proportion of the population carrying *w*Mel falls to 62% when assuming a 10% fitness cost. This is well above what is considered a critical threshold (20–30%) for achieving *w*Mel fixation (100% infection frequency)^29,30^. When dynamics were driven by seven-day moving average temperature inputs, *w*Mel frequency rose faster than with daily inputs; in no instance did frequency fall after reaching fixation.

**Fig. 4:**
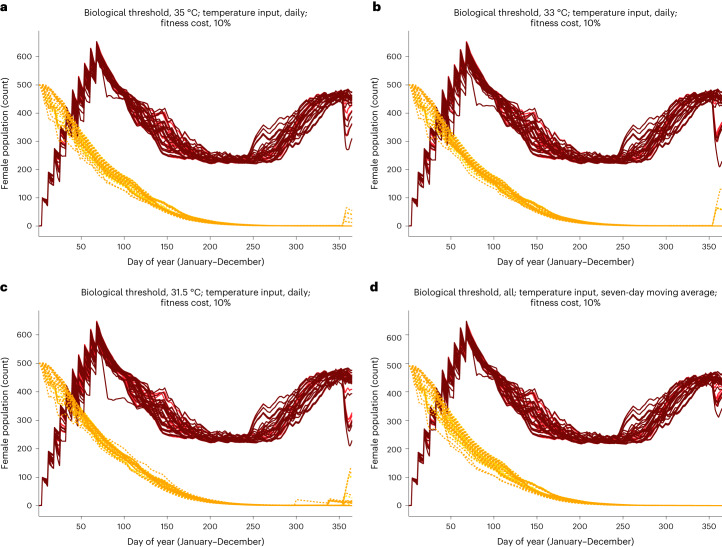
Replacement and suppression dynamics for future heatwave years versus baseline years. Replacement (solid lines) and suppression (dotted lines) dynamics for future heatwave years (darker tones) and the average temperature baselines (lighter tones) from which they are calculated under both future regimes (2030s & 2050s). In each panel, the upper set of trajectories show the *w*Mel-infected population and the lower set of trajectories show the WT population. **a**–**c**, Dynamics given unique thermal thresholds (35 °C (**a**) 33 °C (**b**), 31.5 °C (**c**)) and daily temperature inputs to the *w*Mel dynamic model. **d**, Dynamics when seven-day moving averages are used as temperature inputs; all three thermal thresholds returned equivalent output under this assumption.

The dynamics of *w*Mel-infected and WT female mosquitoes under future regimes put these results into population-level perspective across assumptions of biological threshold and temperature input (Fig. 4). Dynamics occurring under heatwave years were overlaid on dynamics driven by the baseline temperatures used to construct those heatwave inputs, highlighting the delta generated by thermal shocks. The decline in *w*Mel infection frequency and corresponding increase in vector-competent female mosquitoes indicates the potential public health impact of future temperature variability. Using daily temperature inputs, the population of *w*Mel carriers was more than halved by December for selected years in both the 33 °C and 31.5 °C scenarios. A decline was observed for those same years in the 35 °C scenario. In all cases, this decrease corresponded to a rising proportion of vector-competent females.

Current empirical data suggests that *w*Mel falls out of *A. aegypti* at temperatures equal to or exceeding 35 °C (ref. ^22^). In the Cairns climate projections used, this threshold is reached or surpassed as a daily average in 2029 and 2049. While these examples of severe heat have negative effects on simulated *w*Mel interventions (Figs. 3 and 4a–d), they were not sufficient to cause a complete removal of the pathogen-blocking bacterium from the mosquito population. In recent years, temperature in Cairns had greater variability and hotter extremes than the years used as baselines for climate projections^31^ in this location (1986–2005).

Given the robustness of *w*Mel to the Cairns climate scenarios and the potential for warmer or more variable futures in that location and elsewhere, we conducted two additional analyses. First, we undertook a theoretical exploration of what might be required to remove *w*Mel from a population by incorporating progressively hotter days in existing Coupled Model Intercomparison Project Phase 5 (CMIP5) heatwave years (Methods).

**Fig. 5:**
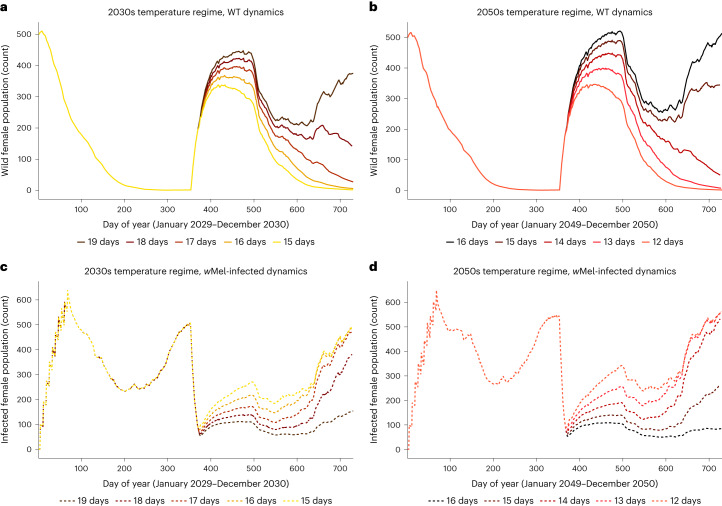
The impact of prolonging heatwaves in future years. The impact of additional hot days on population dynamics in future heatwave years: suppression (solid lines) and replacement (dotted lines) for the 2030s (yellow tones) and 2050s (orange tones). In all panels, darker colors correspond to an increasing number of hot days extending the CMIP5-projected heatwaves. **a**, In the 2030s, less wildtype (WT) suppression is achieved as heatwaves are extended. **b**, Similarly, the 2050s see less WT suppression under longer heatwaves. **c**, *w*Mel replacement is reduced under prolonged heatwaves in the 2030s. **d**, *w*Mel replacement is also lessened given lengthier heatwaves in the 2050s.

We illustrate the effect on the WT population when additional hot days are included in the existing time series of projected future temperatures (Fig. 5a,b). The surge in vector-competent mosquitoes is larger in the 2050 scenario than that of the 2030s due to the high thermal optima of the *A. aegypti* species. Three fewer ‘extra’ days of heat are required in 2050 for the WT population to rebound. However, even under the most extreme assumption of consecutive hot days (19 and 16 in the 2030s and 2050s, respectively), the *w*Mel-infected population persists at levels sufficient to permit a full rebound (Fig. 5c,d).

**Fig. 6:**
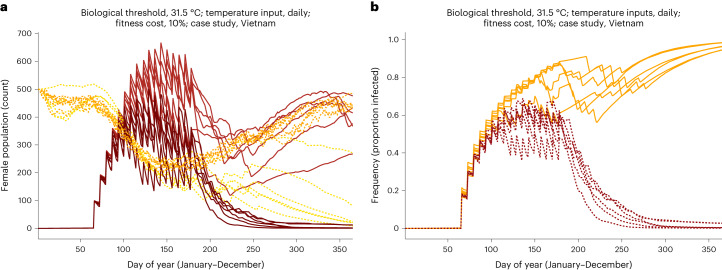
Population dynamics and *w*Mel infection frequency in future years as simulated for the Nha Trang City region of Vietnam. **a**, Replacement (*w*Mel-infected population, solid lines) and suppression (WT population, dotted lines) dynamics during both heatwave years (dark tones) and their baselines (light tones), assuming a 2050 temperature regime. **b**, Effect of future heatwaves on the frequency of *w*Mel infection in an adult female *A. aegypti* population under the 2030 (orange) and 2050 (red) regimes.

In a second investigation of *w*Mel’s thermal limits, we studied data for replacement interventions in the Nha Trang City region, Vietnam. It has hotter historical average temperatures and the potential, according to the CMIP5 projections, for average warming and heatwaves that exceed temperatures and durations predicted for Cairns. Our simulations (Fig. 6) built on the observed environmental conditions and release schedule employed by Hien et al.^16^ in Vinh Luong Ward, where inputs (mean temperature, duration, frequency and amplitude of heatwaves, and intrinsic biological thresholds of *w*Mel) reflect the most extreme assumptions given the data (Methods).

The results show that *w*Mel introgression is generally robust to the tested scenarios as fixation fails only under the most extreme joint assumptions of heatwave duration and intrinsic thermal limit (Fig. 6) (RCP 8.5 2050 climate regimes together with a 31.5 °C or 33.0 °C biological threshold for *w*Mel, more stringent than the empirically demonstrated 35.0 °C point of fallout). This underscores the impact of climate change-induced heatwaves on *w*Mel interventions compared to increases in mean daily temperature: the replacement dynamics driven by the 2050 mean temperatures maintain a high frequency of infection through the end of the period (Fig. 6a, light red solid lines). However, the 2050 heatwaves cause *w*Mel-infected numbers to fall (dark red solid lines). The effect of heatwave duration on sustained fixation is also highlighted: during the 2030s, the longest heatwaves last an average of 9.7 days; in the 2050s, they last 24 days (Fig. 6b).

The Vietnam simulations for the 2030 and 2050 climate regimes reveal an important relationship between infection frequency, heatwaves and the timing of *w*Mel releases. Using daily temperature inputs and a thermal threshold of 31.5 °C, infection frequency was notably increased when intervention releases occurred after the longest heatwave concluded (*P* < 0.0006). However, the average temperature of that heatwave depressed infection frequency (*P* < 0.0001; full model *R*^2^ = 0.8938, corrected Akaike information criterion = 2.1597).

## Discussion

The growing literature on mosquito thermal biology highlights its role in the impact of climate change on vector-borne disease. However, new intervention technologies may also be subject to the complexities of global warming. No research has yet examined the effect of climate change on *Wolbachia*-based biocontrol methods, nor used empirical data on the thermal sensitivity of *Wolbachia* to model the dynamics of this tool. Furthermore, published modelling work that explores the influence of temperature on mosquito-borne disease focuses on trends (increasing average temperatures) not variability (the magnitude, duration and frequency of heatwaves).

In this study, we demonstrate that rising average temperatures in Cairns, Australia under CMIP5-generated predictions may benefit *w*Mel replacement during winter months; however, future summer average temperatures may surpass the symbiont’s thermal thresholds and cause transient population decline. While simulations reflecting the Nha Trang City region of Vietnam reinforce the negative impact of future mean temperatures on *w*Mel replacement under the most stringent assumptions of the bacterium’s thermal thresholds, many non-climatological, non-biological covariates that differ across spatiotemporal settings could also affect the symbiont’s introgression and fixation^16^. This includes the heat-buffering effects of larger or shaded aquatic habitats that can differentially mitigate climate impacts on juvenile microorganisms across built environments, affecting the establishment and persistence of both *w*Mel and their *A. aegypti* hosts^16,32^.

Our results exhibit the potential vulnerability of *w*Mel-based replacement to temperature variability under climate change as simulations with hotter and more frequent thermal extremes show diminished efficacy relative to simulations that strictly consider mean temperature. CMIP5-projected heatwaves in Cairns lower *w*Mel frequencies; however, frequencies recover under even the most severe projections of local heat shocks. Outputs for the 2030 scenarios in the Nha Trang City region bolster the finding that *w*Mel technology may withstand near-term future periods of extreme temperature, suggesting that our conclusions can be extrapolated to additional geographical contexts.

In 2018 (26–28 November), a heatwave in Cairns where *w*Mel infection had been established since 2011 presented a natural experiment that produced findings aligning with ours^33^. Recorded maximum temperatures reached 43.6 °C, and while *w*Mel frequency fell, it never dropped below 83% in juvenile mosquitoes. It recovered to nearly 100% by April 2019. However, throughout this period, the highest average daily temperature was 33.75 °C. To explore the edge case in which *w*Mel is severely reduced or eliminated from a population after heat shocks, empirical experimentation should assess the effect of a daily average greater than or equal to 35 °C, and evaluate the interaction of temperature spikes with mosquito developmental stages on pathogen-blocking efficacy^34^.

Increasingly acute and prolonged heatwaves around the world highlight the need to test the thermal limits of *w*Mel technology beyond the theoretical examples presented in this article, where approximately two to three weeks at or above 35 °C reduced *w*Mel frequency during the examined period. Simulation results for the 2050 heatwaves in the Nha Trang City region show *w*Mel elimination under the lower thresholds tested (31.5 °C, 33.0 °C). While such dynamics may be relevant to a proposed toxin–antidote gene drive based on the *Wolbachia* alleles responsible for CI^35^, they also highlight a possible 30-year time horizon in which to advance the heat tolerance of current *w*Mel-based replacement technology.

Further investigation is required to discern the minimal exposure time at or above *w*Mel’s biological threshold before critical mechanisms such as CI and maternal inheritance are impacted, and to understand the generational duration of deleterious effects after heat spikes. The vector–pathogen relationship under heat stress also merits more study: dengue virus increases the thermal sensitivity of *A. aegypti*, and coinfection with *Wolbachia* does not furnish protection from the dengue-induced thermotolerance effects^36^. However, there is evidence that diurnal temperature fluctuations negatively impact the vector–pathogen relationship at mean temperatures greater than 18.0 °C, with *A. aegypti* having shorter lifespans and being less likely to become infected with dengue virus as the diurnal temperature range increases^37^.

Our modelling omits the potential heat avoidance of mosquitoes^38,39^ and thus the possibility that *Wolbachia*-infected *A. aegypti* may seek cooler temperatures where bacterial titre is maintained^38^. Such heat-induced behaviour^14^, as well as the connection between *Wolbachia* density and CI mechanisms^40^, require further research. Finally, our results are necessarily a function of structural decisions made when developing the ordinary differential equation (ODE) model, including the choice of functions to define temperature-sensitive vital rate parameterizations^41^. Therefore, analyses using alternative formulations may also be merited.

*Wolbachia* experts recognize that strain selection must account for local environmental realities^14^. Recent studies focused on deployment in extreme environments^42^ and the development of strains with increased heat tolerance^43^. Field trials across an increasing variety of geographies will furnish critical data on the effect of climate and non-climate drivers on *w*Mel introgression and persistence. For decision-makers to weigh the choice between *w*Mel and its alternatives as we move into an uncertain and more variable climate future, computational and bench scientists should explicitly account for predictions of regional warming—particularly heat extremes—in their experimental designs. In the short term, temperature-conscious operational planning, such as augmented deployment schedules, may be able to compensate for the already observed risk that infection frequencies drop or remain at low levels due to heat stress.

While the results presented in this article furnish reassurance that *w*Mel replacement is a resilient technology in the face of near-term climate change, they are predicated on temperature profiles constructed from pre-2006 baselines. Thus, they remain conceivably best-case scenarios, given the virtual certainty that hot extremes will continue to increase in both frequency and intensity^44^. This highlights the inextricable link between climate mitigation policy and infectious disease management. Failure to curb carbon dioxide emissions will not only increase the proportion of the world exposed to deadly mosquito-borne illnesses due to rising temperatures; it may also undercut the utility of an otherwise demonstrably effective tool for vector control.

## Methods

### Climate data for Cairns, Australia

We obtained historical daily temperature data for Cairns, Queensland, Australia (latitude 16° 52' 25.9594" S, longitude 145° 44' 44.88" E) from the Global Historical Climatology Network database maintained by the National Centers for Environmental Information of the United States National Oceanic and Atmospheric Administration (NCEI–NOAA). We selected Cairns as the geographical location of interest because two towns in that region, Yorkeys Knob and Gordonvale, were the sites of the first successful field trial for population replacement using *w*Mel; therefore, the reported trial outcomes serve as experimental validation for simulations using local environmental variables^10^. Additional *Wolbachia* releases have since taken place in the area, including in central Cairns^17,18,45^.

Projected future data for Cairns, including both average temperatures and heatwaves under the RCP scenarios 4.5 and 8.5, were developed from dynamically downscaled CMIP5 global climate projections obtained from the Queensland Future Climate Dashboard, which is maintained by the Science Division of the Queensland Department of Environment and Sciences^46,47^. These data are recorded in fractions of degrees of long-term change (°C) relative to the reference period (1986–2005). Because the available daily historical temperature data begin in 1990, we used the period 1990–2005 as the historical baseline and developed daily future climate scenarios using the anomaly method for the years 2024–2059. While the years from 2060 onwards exhibit more drastic temperature rise, we excluded these from the analysis to focus on outcomes most relevant to near-term technological decisions concerning *Wolbachia*-based public health interventions. Full details on the methodology used to construct future daily average temperatures and heatwaves are shown in section 2 of the Supplementary Information.

### Climate data for Nha Trang City, Vietnam

We sourced historical observed daily temperature records for the region of Nha Trang City (Nha Trang), Vietnam (latitude 12° 15' 12.2394" N, longitude 109° 11' 13.56" E) from the NCEI–NOAA. We chose this location given its higher average baselines and longer, more frequent and hotter future heatwaves compared to the Cairns case study. Furthermore, two villages in the Nha Trang region, Vinh Luong Ward (Vinh Luong) and Tri Nguyen Village (Tri Nguyen), are sites of recent *w*Mel-infected *A. aegypti* releases. The field study associated with these trial interventions used weather data from Nha Trang to conduct subsequent analysis given the absence of such data from these two smaller nearby population centres^16^. Future average temperature data for Vietnam under RCP scenario 8.5 were sourced from the Climate Change Knowledge Portal^48^; future heatwave data were furnished by Dong et al.^49^. Both projected mean and heatwave scenarios were produced using historical temperature data for the baseline period (1990–2005) and the anomaly method described above. For additional details, see section 2 of the Supplementary Information.

### Simulation model of population dynamics

We developed a system of ODEs to simulate mosquito population dynamics. The model, described in detail in section 3 of the Supplementary Information, uses a daily time step and portrays four life stages of the mosquito lifecycle: egg, larva, pupa and adult^50,51^. Adults emerging from the pupal stage are evenly divided between males and females; the population is assumed to be randomly mixing and all organisms within a particular life stage are equal with respect to their birth, death and maturation rates. Larval stage mortality is modulated by logistic density dependence^52^. Parameterization reflects the species-specific thermal biology of *A. aegypti*, which was selected for this study because it has been a primary host for *w*Mel in the context of population replacement experiments including in the Cairns and Nha Trang regions. The model was initialized at equilibrium. The equilibrium of a dynamic system generated by a system of ODEs is defined as a solution that does not change over time; it is a steady state.

*A. aegypti* vital rates were calculated using temperature-responsive functional forms unique to each life stage. These formulations and their parameterization were developed by Rossi et al.^53^ to probe the effect of climate change on the *A. aegypti* life cycle and its relevance to disease incidence. The assumptions used to adapt these equations to the underlying population model included using literature-derived values for oviposition (63 eggs daily per female) and excluding the consideration of carrying capacity for egg laying^54^. Field studies of *w*Mel replacement interventions in the Cairns region informed the deployment schedule used for the simulations using climate inputs from that geographical location, where the frequency of model releases paralleled reality to occur every seven time steps from day 4 through day 63 (ref. ^10^). The interventions modelled using the Nha Trang temperature data used a schedule that emulated studies conducted in nearby Vinh Luong, with releases taking place every seven days from day 65 through day 177 (ref. ^16^). The Vinh Luong trials furnished the basis of our experimental design because interventions at that location occurred during one of the hottest years on record for the region (2018).

### Biological parameterization of *w*Mel temperature sensitivity

*Wolbachia* carriers in this work are assumed to have temperature-responsive hatch rates, maternal inheritance rates and CI levels. Empirical data generated from the cyclical heat exposure of *w*Mel-infected *A. aegypti* mosquitoes was used to develop the equations that reflect thermal sensitivity^22^. In these laboratory experiments, eggs were subjected to weekly temperature regimes that cycled daily between the maximum and minimum of a given 10 °C range, with the coolest regime averaging 29 °C and the warmest averaging 37 °C. Data were also collected for eggs held at a consistent 26 °C for the seven-day study period.

Extrapolating the results of week-long experiments to a dynamic model with daily time steps requires assumptions about the unknown functional form of heat stress accumulation in *w*Mel. To address this uncertainty and account for the lack of required temporal resolution in the empirical data-generating process, we included a sensitivity analysis that assesses two theoretical alternatives: first, all temperature inputs to the thermally sensitive *w*Mel equations were calibrated to consist of a seven-day moving average. This reflects an assumption that the physiological impact of heat stress is evenly distributed, with the effect of one hot day prolonged for a subsequent six days. In the second sensitivity analysis, all temperature inputs were allowed to be entered as daily values. This reflects an assumption that the impact of heat stress is primarily immediate and does not necessarily result in a cumulative effect within the organism.

To formulate the biological model of *w*Mel, we fitted a function to the mean value of experimental replicates for each temperature regime. Equation (1) reflects the statistical best fit to egg hatch data. The hatch rate *T(C)* for infected eggs below 26 °C was fixed at 0.91833; above 36 °C, it was fixed at 0 in accordance with Ross et al.^22^. Supplementary Fig. 1 compares the equation (1) dynamics with the observed hatch rates:

1 $$T(C)=\left\{\begin{array}{ll}0.918 & {{\mathrm{if}}}\,C\le 26\\ 0.916-0.020(C-26)-0.006{e}^{0.474(C-26)} & {{\mathrm{if}}}\,26 < C\le 36\\ 0 & {{\mathrm{if}}}\,C > 36\end{array}\right.$$

The temperature-sensitive status of *w*Mel infection and consequent vertical transmission from mother to offspring was modelled using matrix operations informed by the data underpinning Figs. 7b,c of Ross et al.^22^, where above 35.0 °C *w*Mel inheritance, *I*, falls to 0 and previously complete CI, *Y*, stops functioning entirely. These conditional relationships are formalized in equations (2) and (3):

2 $$I(C)=\left\{\begin{array}{cc}1.0 & {\rm{if}}\,C < 35\\ 0 & {\rm{if}}\,C\ge 35\end{array}\right.$$

3 $$Y(C)=\left\{\begin{array}{cc}1.0 & {\rm{if}}\,C < 35\\ 0 & {\rm{if}}\,C\ge 35\end{array}\right.$$

The conservative assumptions embodied by this approach, which rely on the simple presence or absence of *w*Mel infection, are made in acknowledgement of the fact that the mappings between infection titre and the biological properties it confers are not yet well defined. The chosen method results in a probable overestimation of both *w*Mel maternal transmission rates and CI at temperatures lower than the 35.0 °C threshold. Therefore, we include additional sensitivity analyses varying this biological threshold, imposing it instead at 31.5 °C and 33.0 °C. Future work, including empirical experimentation to improve scientific understanding of the temperature sensitivities of *w*Mel infection and the mechanisms of CI, will enable refined mathematical estimations of these processes.

A range of fixed fitness costs was modelled to reflect heightened mortality in *w*Mel carriers, drawing on empirically derived values in the literature. This sensitivity analysis tested costs that augmented adult mortality rates in *w*Mel-infected mosquitoes by 0%, 10% and 20%, with reported results reflecting use of the middle value (10%). Section 5 of the Supplementary Information contains outcomes using the upper and lower bounds of the fitness range. Field studies of *w*Mel replacement interventions in the Cairns region are consistent with model dynamics, as illustrated in Supplementary Fig. 2 comparing observed data with simulated results (SupplementaryInformation).

### Metrics used to assess and compare simulation results

In addition to characterizing the results using qualitative visual comparisons, a new standard entomological metric (SEM) is proposed; it was used to evaluate the replacement capacity of *w*Mel-based interventions under past and future climate scenarios, that is, the RES. Like the SES put forward in Vásquez et al.^28^, the RES is a single value that summarizes relative success over the period of interest, and can be used to compare the performance of alternative interventions to each other. The RES is assessed with respect to the frequency of *Wolbachia* infection in the standing vector population over this time. A conceptual explanation of the score is furnished by Supplementary Fig. 3 (see section 1 as well as section 4 of the SupplementaryInformation; the latter includes illustrative example applications).

There are a total of *T* time steps in the intervention period being evaluated; these are equal to that period’s length, *τ*_f_ − *τ*_0_, divided by the discretization, *δτ*. This is explained in equation (4):

4 $$T=\frac{{\tau }_{\mathrm{f}}-{\tau }_{0}}{\delta \tau }$$

The frequency of *w*Mel is estimated by calculating the proportion of the infected female population *P*_*g,t*_ and integrating the cumulative change in that population over the duration of an intervention. $A^F_{g,t}$, the area under the curve of the observed frequency trajectory, is defined in equation (5):

5 $${A}_{g,t}^{F}=\frac{({P}_{g,t-1}+{P}_{g,t})}{2}\delta \tau$$

This area is then divided by the outcome of an idealized intervention *Γ*_*g*_ wherein fixation (100% infection of the standing population) is achieved immediately and for the duration of the period of interest; this yields the RES score described by equation (6). Like the previously defined SEMs, indexing the RES score *R*_*g*_ and vector population change *A*^*F*^_*g,t*_ according to patterns of inheritance *g* allows the metric to be generalized beyond *w*Mel-specific applications. When evaluating public health intervention technologies that use genetic modification, *g* is used to represent the frequency of the refractory genotype rather than *Wolbachia* infection status:

6 $${R}_{g}=\frac{{\sum }_{t=1}^{T}{A}_{g,t}^{F}}{{\varGamma }_{g}}$$

### Theoretically testing the thermal limits of *w*Mel

Using 2029 RCP 8.5 and 2049 RCP 8.5 as examples, we augmented each time series of temperature inputs to include, beginning on day 355, consecutive additional days of heat equivalent to the maximum value in that time series. For 2029, the maximum value was 35.21 °C and for 2049 it reached 36.6 °C. These temperatures are high enough to drop maternal transmission of *w*Mel and hatch rates for *Wolbachia*-infected eggs to 0. CI for crosses between wild females and *w*Mel-carrying males is also eliminated. We selected the first time step to begin appending new hot days based on the culmination of the heat surge in the 2029 example, which ended on day 354. To maintain comparability, the same time step was chosen for the 2049 simulation. The time horizon was then extended by a second year and no additional *w*Mel-infected releases were conducted.

## Online content

Any methods, additional references, Nature Portfolio reporting summaries, source data, extended data, supplementary information, acknowledgements, peer review information; details of author contributions and competing interests; and statements of data and code availability are available at 10.1038/s41558-023-01746-w.

### Supplementary information

Supplementary Information Supplementary Methods, Discussion, Figs. 1–8 and Tables 1–27.

## Data Availability

The raw data outputs featuring the population dynamics that resulted from all model runs is stored on Figshare: 10.6084/m9.figshare.20722270; 10.6084/m9.figshare.20721928; 10.6084/m9.figshare.20719234; 10.6084/m9.figshare.20716117; 10.6084/m9.figshare.20607765; 10.6084/m9.figshare.22589623; 10.6084/m9.figshare.22589626; 10.6084/m9.figshare.22589632 (refs. ^55–62^).

## Code availability

The GeneDrive.jl software extended to implement this work is available via GitHub at https://github.com/vnvasquez/GeneDrive.jl (ref. ^63^). All code adaptations required to reproduce the specific experiments in this paper are available via GitHub at https://github.com/vnvasquez/WolbachiaClimatePaper (ref. ^64^).
