# Publisher Correction: *w*Mel replacement of dengue-competent mosquitoes is robust to near-term climate change

**DOI:** 10.1038/s41558-023-01797-z

**Published:** 2023-08-14

**Authors:** Váleri N. Vásquez, Lara M. Kueppers, Gordana Rašić, John M. Marshall

**Affiliations:** 1 Energy and Resources Group, University of California, Berkeley, CA USA; 2 Department of Electrical Engineering and Computer Sciences, College of Engineering, University of California, Berkeley, CA USA; 3 Climate and Ecosystem Sciences Division, Lawrence Berkeley National Laboratory, Berkeley, CA USA; 4 Mosquito Genomics, QIMR Berghofer Medical Research Institute, Brisbane, Queensland Australia; 5 Divisions of Epidemiology and Biostatistics, School of Public Health, University of California, Berkeley, CA USA; 6 Innovative Genomics Institute, University of California, Berkeley, CA USA

**Keywords:** Ecological epidemiology, Ecological modelling

Correction to: *Nature Climate Change* 10.1038/s41558-023-01746-w. Published online 3 August 2023.

In the version of this article initially published, the word “climate” was missing from the article’s title; the error has been corrected in the HTML and PDF versions of the article.

