# Supplementary material for: wMel replacement of dengue-competent mosquitoes is robust to near-term climate change
Source: Nat Clim Chang. 2023 Aug 3;13(8):848–55. doi: 10.1038/s41558-023-01746-w (PMC10403361; doi:10.1038/s41558-023-01746-w)
Supplement: Supplementary file 1 — Supplementary Methods, Discussion, Figs. 1–8 and Tables 1–27. [file 41558_2023_1746_MOESM1_ESM.pdf]

nature climate change

Article

https://doi.org/10.1038/s41558-023-01746-w

*w*Mel replacement of dengue-competent mosquitoes is robust to near-term climate change

In the format provided by the authors and unedited

Section 1: Methods Figures

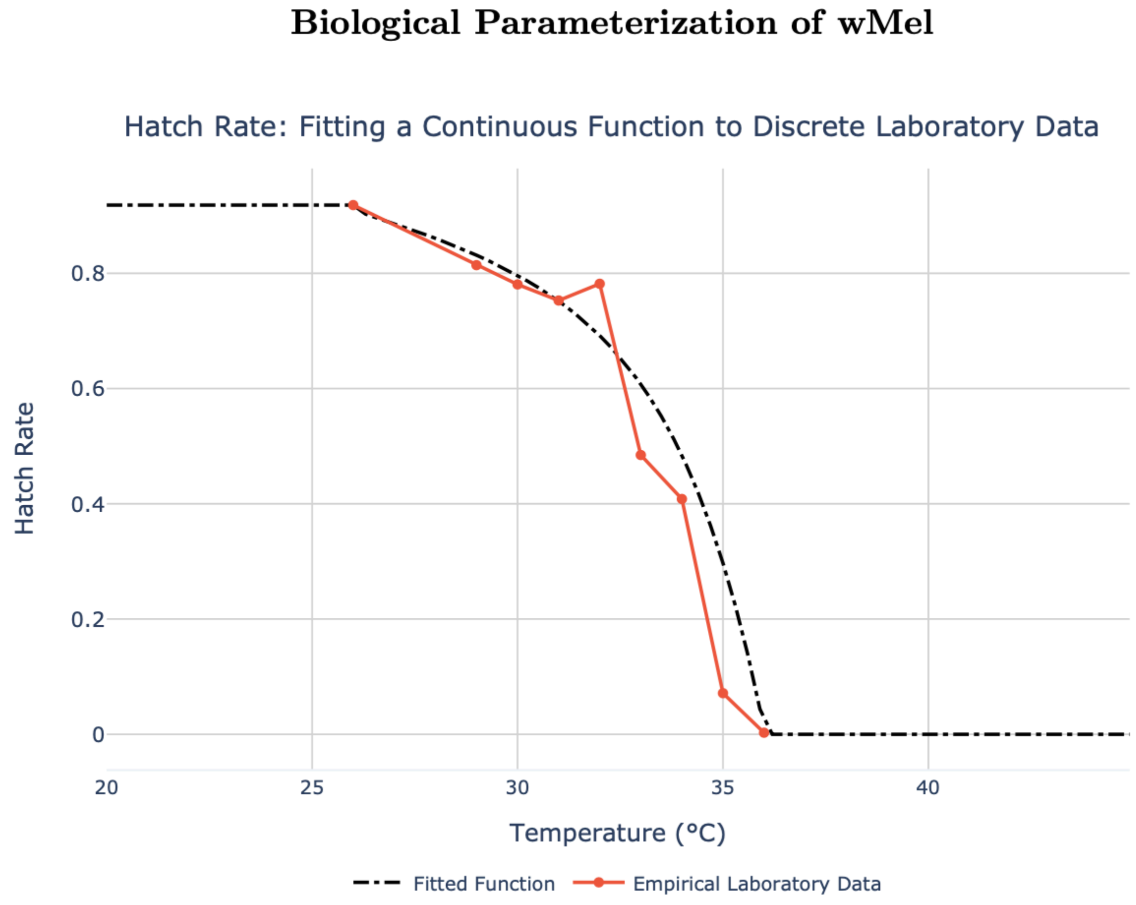

Supplementary Fig. 1: Fitted hatch rate function (Equation 1) plotted against empirical data underlying Figure 7a of Ross et al (2019), which recorded hatch rates for wMel-infected eggs under cyclical temperatures.

Validating Model Output with Field Trial Results

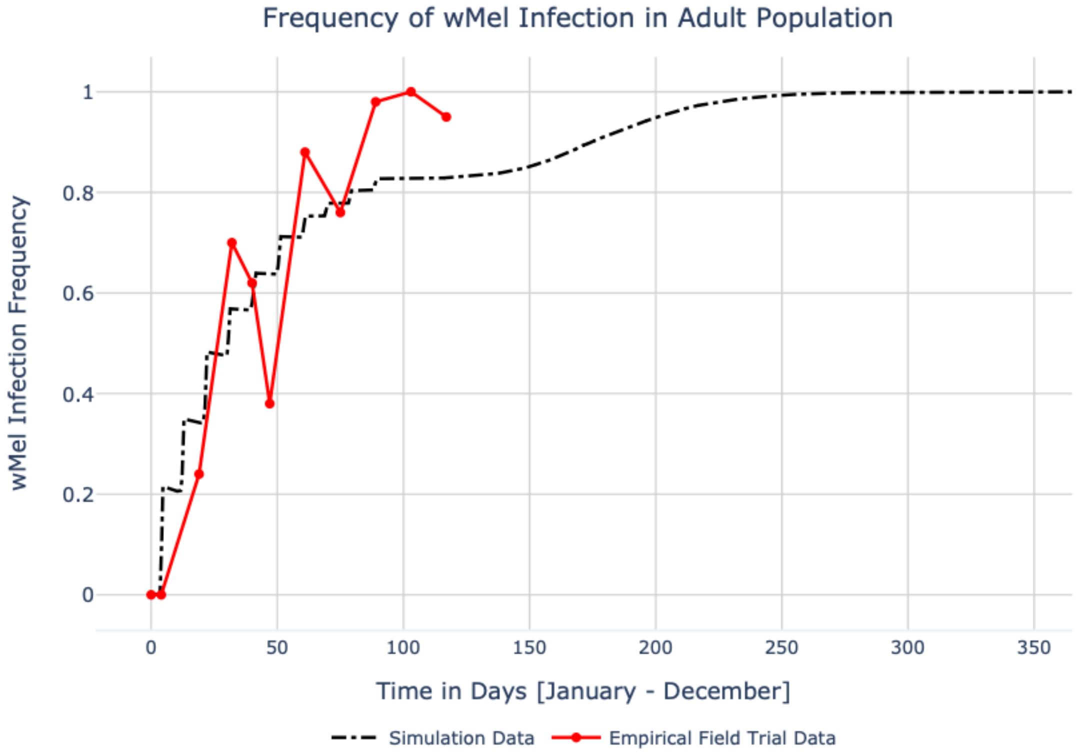

Supplementary Fig. 2: Empirical results from a 2011 field trial in Yorkeys Knob, Queensland, Australia support model dynamics. Here, a wMel-based replacement intervention with a release schedule based on that of the field trial is simulated using 2011 recorded temperatures, and results are compared with observed data. Figure 1A of Hoffman et al (2011) furnished the field observations used here. The final day of data collection in the field trial was April 27 (day 117); modelled results are shown through December 31 (day 365).

Visual Explanation of the Replacement Efficacy Score Metric

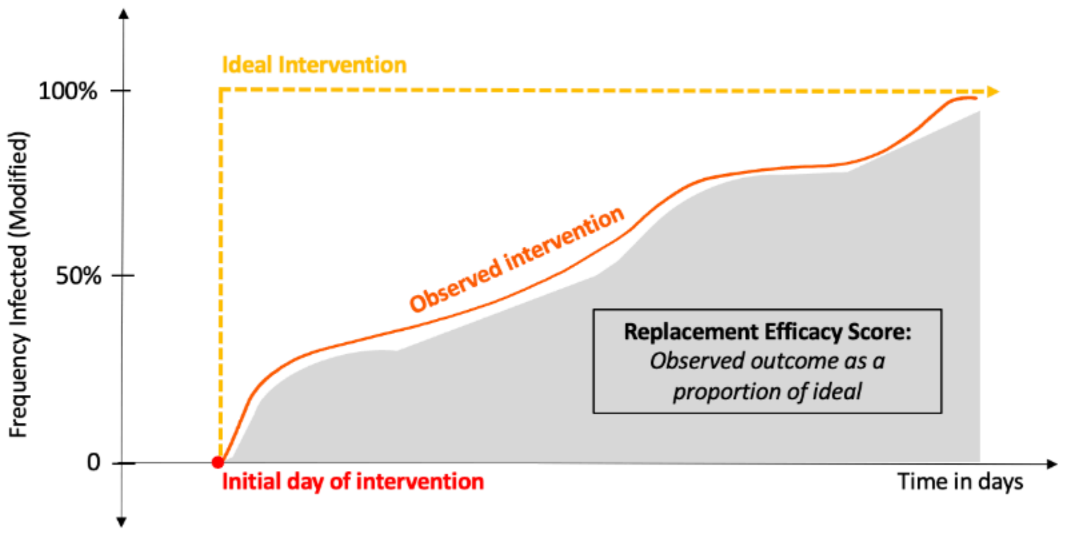

Supplementary Fig. 3: The Replacement Efficacy Score (RES) reflects the proportional comparison between the ideal and observed intervention outcomes with respect to the frequency of the modified population over the period of interest. Modification, in the case of wMel, refers to infection with the symbiont.

Section 2: Synthesized Climate Data

<u>*Cairns, Australia*</u>

The freely available Global Historical Climatology Network (GHCN) database is a composite of climate records merged from local sources and subjected to quality assurance review; the specific records employed for this study that serve as the historical baseline were sourced from Cairns Aero (Meteorological Station ID: ASN00031011) and include date as well as daily average, maximum, and minimum temperatures for January 1, 1990, through December 31, 2019.

To produce future daily average temperatures for both RCPs, the deltas in degrees Celsius corresponding to each projected month and year from the QFCD dataset for the Queensland region were added to the historical baseline creating a single future time-series of the same

length and with the same variability as the historical time-series. The future heatwave scenarios were constructed by first identifying historical heatwaves in the 1990-2019 dataset, using the Australian Bureau of Meteorology's definition: "a period of at least three days where the combined effect of excess heat and heat stress is unusual with respect to the local climate."[1,2]

We defined "unusual with respect to the local climate" as daily maximum temperatures that exceeded the historical average daily maximum temperature for the corresponding day of year by at least three degrees Celsius for at least three consecutive days. Six years within the 15-year historical baseline period containing days that met these requirements were selected: 1990, 1992, 1994, 1995, 2002, and 2005. Future heatwaves were created for the corresponding years in the future time series – specifically for 2024, 2026, 2028, 2029, 2036, and 2039 as well as 2044, 2046, 2048, 2049, 2056, and 2059. The average daily temperature for the corresponding future date of each heatwave identified within the historical record was augmented by the change in °C recorded by the "Heatwave Peak Temperature" variable in the QFCD dataset.

The duration of each heatwave identified within the historical record was also lengthened in the future time-series according to the change in days specified by the "Heatwave Duration" variable in the QFCD dataset, where each fraction of a day was rounded to the nearest full day. The additional heatwave days were given the same temperature delta in °C indicated by the "Heatwave Peak Temperature" variable, on top of the future average temperature for that date.

Finally, to reflect scientific projections of future heatwave frequency – the change in the number of heatwave days each year – every future heatwave year was augmented with an increase in the total count of heatwave days corresponding to the "Heatwave Frequency" variable in the QFCD dataset. This variable carries a distinct value for each season (wet vs. dry), each RCP scenario (4.5 vs. 8.5) and each future set of years (2030 vs. 2050). To designate a new heatwave day with consistent logic and accurate implementation while accounting for all constraints, including the requirement that "heatwaves" be defined as three consecutive days where the average daily maximum temperature meets or exceeds the average historical baseline temperature for the corresponding day of year by 3°C, a simple algorithm was developed.

For each season of each heatwave year, a running three-day average was taken of the daily maximum temperatures. These averages were sorted, greatest to least, according to the delta by which they exceeded the historical baseline for the corresponding day of year. Any day already defined as a historical heatwave day was dropped from the tally of running averages and excluded from this ordering. The N hottest averages, where N = (Heatwave Frequency)/3, that were at minimum four days apart from each other, were designated as new future heatwave days. The average daily temperature of the newly selected days, together with the two adjacent days that together informed the qualifying three-day running averages, was then augmented by the value of the "Heatwave Peak Temperature" variable.

Supplementary Figure 4 features a temporal subset of four timeseries created using this methodology for the years 2029 and 2049 from the historic baseline of 1995. It illustrates the differential between 2030 and 2050 temperature regimes for both RCP Scenarios 4.5 and 8.5 in the case of heatwaves as well as average temperature.

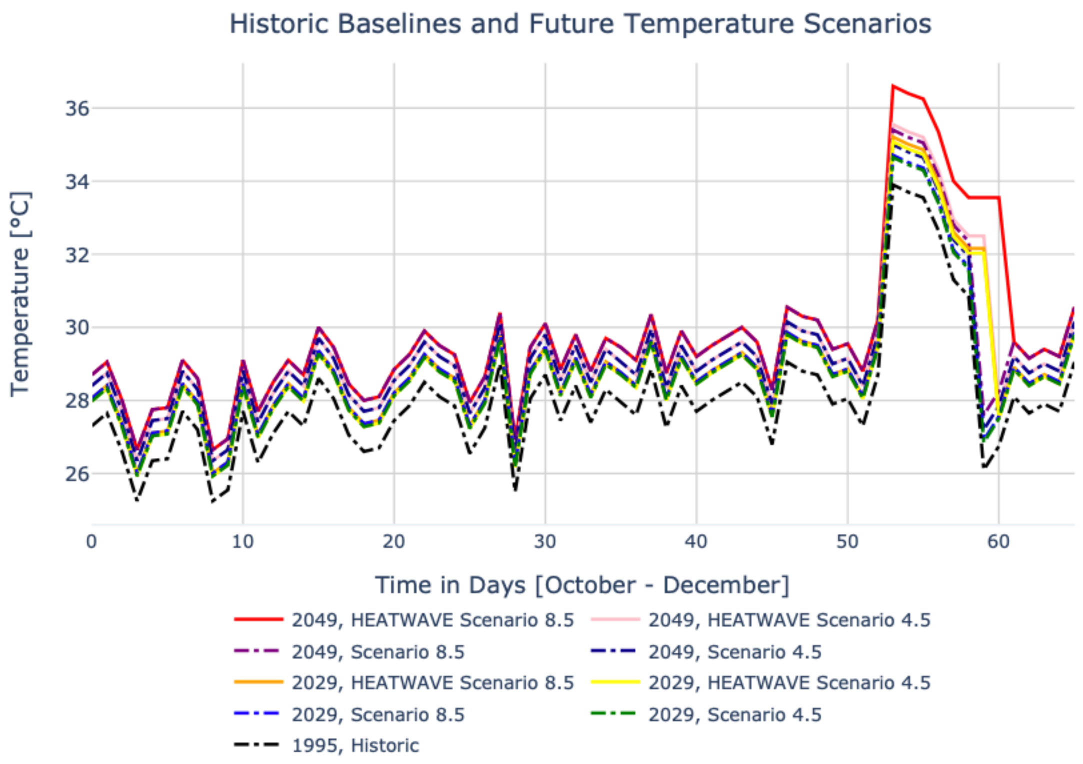

Supplementary Fig. 4: Subset of example temperature time series developed using RCP 4.5 and 8.5 scenarios.

Nha Trang, Vietnam

As above, the observational daily records that serve as the historical temperature baseline for the region of Nha Trang City, Vietnam (Nha Trang) were sourced from NCEI-NOAA, using Meteorological Station ID: 48877099999, for January 1, 1990, through December 31, 2019. We obtained CMIP5 projections of temperature deltas for Vietnam under 2030 and 2050 climate regimes for RCP 8.5 from the World Bank Climate Change Knowledge Portal.[3] Following the established anomaly method, we used these together with the historical baseline to produce timeseries of future daily average temperatures.

Because the observed dataset for Nha Trang did not contain any periods conforming to the Australian Bureau of Meteorology's definition of heatwaves, we developed future heatwave years as follows. First, we subset the 1990-2019 temperature records to isolate those years which were both within the designated CMIP5 baseline period (1986-2005) and contained at least one day of maximum temperature exceeding the historical average daily maximum for the corresponding day of year by at least three degrees Celsius. We further subset this data to isolate the six (to use the same number of heatwave years examined for Cairns) remaining years with the hottest annual average temperature: 1997, 1998, 2001, 2002, 2003, and 2005.

With these baselines, we constructed future heatwaves using a modified iteration of the algorithm described in the previous section: for each historical year, a running three-day average was taken of the daily maximum temperatures. These averages were sorted, greatest to least, according to the delta by which they exceeded the historical baseline for the corresponding day of year. The N hottest averages, where N = (Heatwave Frequency - X)/3 + 1 and X = the duration of the longest projected heatwave in days, were designated as new future heatwave days. The daily temperature values of the full year were updated to reflect future average mean temperatures, after which the temperature of the newly selected heatwave days was augmented by the value of the "Heatwave Peak Temperature" variable in Dong et. al. (2021). The warmest of the N hottest running averages identified within the historical record was extended in the future time-series according to the "Heatwave Duration" variable in the Dong et. al. (2021) dataset, where each fraction of a day was rounded to the nearest full day. The total number of heatwave days was constrained to equate to the "Heatwave Frequency" variable in the Dong et. al. (2021) dataset.

Section 3: Mosquito Population Model

The model equations representing the change in numbers of *Ae. aegypti* for the various life stages within a single geographic node are as follows. They assume a timestep of one day; all simulations run using them in this work are on a time horizon of one year unless otherwise noted.

$$\omega_g = \sum_{i=1}^{N} \beta_g \sigma_g \left(\mathbf{\Gamma}_g \odot \mathbf{T}_g\right)_i F_i \qquad \forall g \qquad (1a)$$

$$\frac{dE_{g,1}}{dt} = \omega_g - E_{g,1}(\mu_E + q_E n_E) \qquad \forall g \qquad (1b)$$

$$\frac{dE_{g,i}}{dt} = E_{g,i-1} q_E n_E - E_{g,i}(\mu_E + q_E n_E) \qquad \forall g, i = 2 \dots n_E \qquad (1c)$$

$$\frac{dL_{g,1}}{dt} = E_{g,n_E} q_E n_E - L_{g,1}(\mu_L d + q_L n_L) \qquad \forall g \qquad (1d)$$

$$\frac{dL_{g,i}}{dt} = L_{g,i-1} q_L n_L - L_{g,i}(\mu_L d + q_L n_L) \qquad \forall g, i = 2 ... n_L \qquad (1e)$$

$$\frac{dP_{g,1}}{dt} = L_{g,n_L} q_L n_L - P_{g,1}(\mu_P + q_P n_P) \qquad \forall g \qquad (1f)$$

$$\frac{dP_{g,i}}{dt} = P_{g,i-1} q_P n_P - P_{g,i}(\mu_P + q_P n_P) \qquad \forall g, i = 2 ... n_P \qquad (1g)$$

$$\frac{dm_g}{dt} = P_{g,n_P} q_P n_P (1 - \theta_g) - m_g \mu_m \qquad \forall g \qquad (1h)$$

$$X_g = P_{g,n_P} q_P n_P \theta_g \frac{m_g \eta_g}{\sum_{k=1}^{N} m_k \eta_k} \qquad \forall g \qquad (1i)$$

$$\frac{dF_{g,i}}{dt} = X_{g,i} - F_{g,i} \mu_F \qquad \forall g, i \qquad (1j)$$

Here, the number of individuals in juvenile stages of egg, larva, and pupae are represented by variables *E*, *L*, and *P* and distinguished by genotype *g*, which reflects a particular pattern of inheritance. In this work, *g* is used to differentiate wildtype and *wMel*-infected individuals. Adult stages male, *m*, and female, *F*, are assumed to mate once immediately upon emergence from the pupal stage. Males are referred to using a lowercase letter because, unlike the juvenile or female stages, this stage is implemented as a vector rather than a matrix. Mated females are represented by *X*. The index *i* in this notation indicates time in days. To initialize the model at equilibrium,

the left hand side of the ODE system is set to zero and solved; the results of this process are then verified using a numerical software package (NLSolve.jl).

Wildtype mortality rates μ and development rates q are dynamically calculated according to temperature using the functional forms specified by Rossi et al (2014).[4] Fitness cost is implemented as a 0%, 10%, or 20% increase in μ of both adult stages. *Wolbachia*-infected mortality rates μ, inheritance probabilities Γ_g, and survival probabilities T_g are dynamically calculated according to temperature as described in the Methods section of this work.

Logistic density dependence d is implemented in the larval stage L. The number of eggs laid per genotype is represented by ω_g, while β_g and σ_g are female and male fecundity parameters and Γ_g and T_g convey the inheritance and survival probability of the specified genotypes, respectively. The parameterization of Γ_g follows Sánchez et al (2020)[5] while the formulation of Equations 1a and 1i follow Sánchez et al (2020).[6]

Section 4: Replacement Efficacy Score

The Replacement Efficacy Score (RES) concept is designed as a summary metric useful for comparing the replacement achieved by alternative scenarios relative to each other, rather than being necessarily an absolute indicator of success or failure in and of itself. The value of the RES score is contingent on the dynamics observed over the course of the period of interest.

To illustrate, we see in the hypothetical example below how failure to fixate can produce distinct RES scores even in the context of the same intervention scheme featuring a single release of the same size into a standing wildtype population of the same size (**Supplementary Figure 5**).

In juxtaposing these two RES values, we can deduce that the replacement effort was 29% more successful under scenario A relative to scenario B and proceed to analyze the situational differences (e.g., baseline temperature, temperature variability, differences in release schedules).

Here, the delta in RES stems from the temperature difference between the two scenarios, where the warmer scenario B would require a larger intervention (whether via the size of the individual release or a greater frequency of releases) to achieve a RES score that is equivalent to the cooler scenario A.

Examples to Illustrate the Replacement Efficacy Score Metric

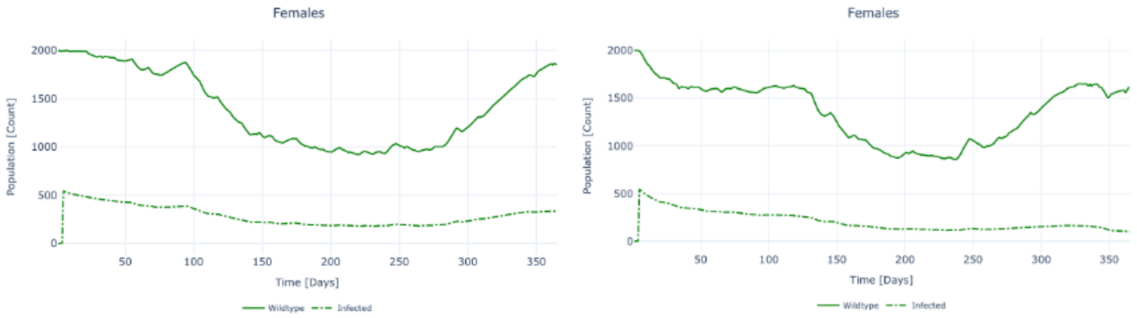

(a) (b)

Supplementary Fig. 5: Visualizations of the two sets of population dynamics that produce alternative Replacement Efficacy Scores (RES). Panel (a) shows Scenario A dynamics, the product of observed temperature inputs from 2011 in Cairns, Australia. The RES calculated for Scenario A is **16.8**. Panel (b) shows Scenario B dynamics, where temperature inputs are comprised of 2011 daily values plus a 2°C perturbation. Scenario B produces a RES of **13.0.**

Section 5: Additional Results and Sensitivity Analyses

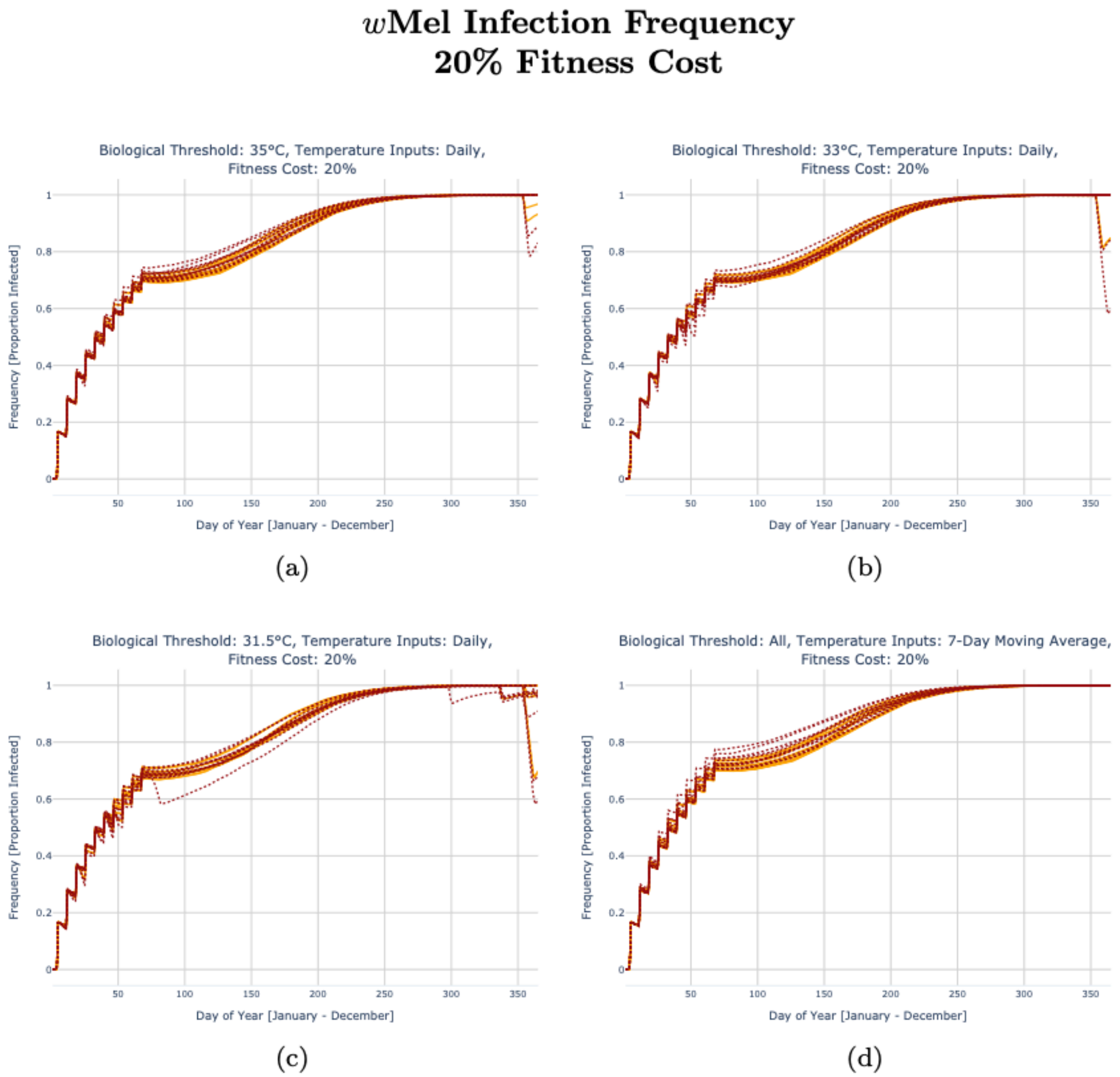

Supplementary Fig. 6: Effect of future heatwaves on wMel infection frequency under Cairns 2030 & 2050 climate scenarios, assuming 20% fitness cost.

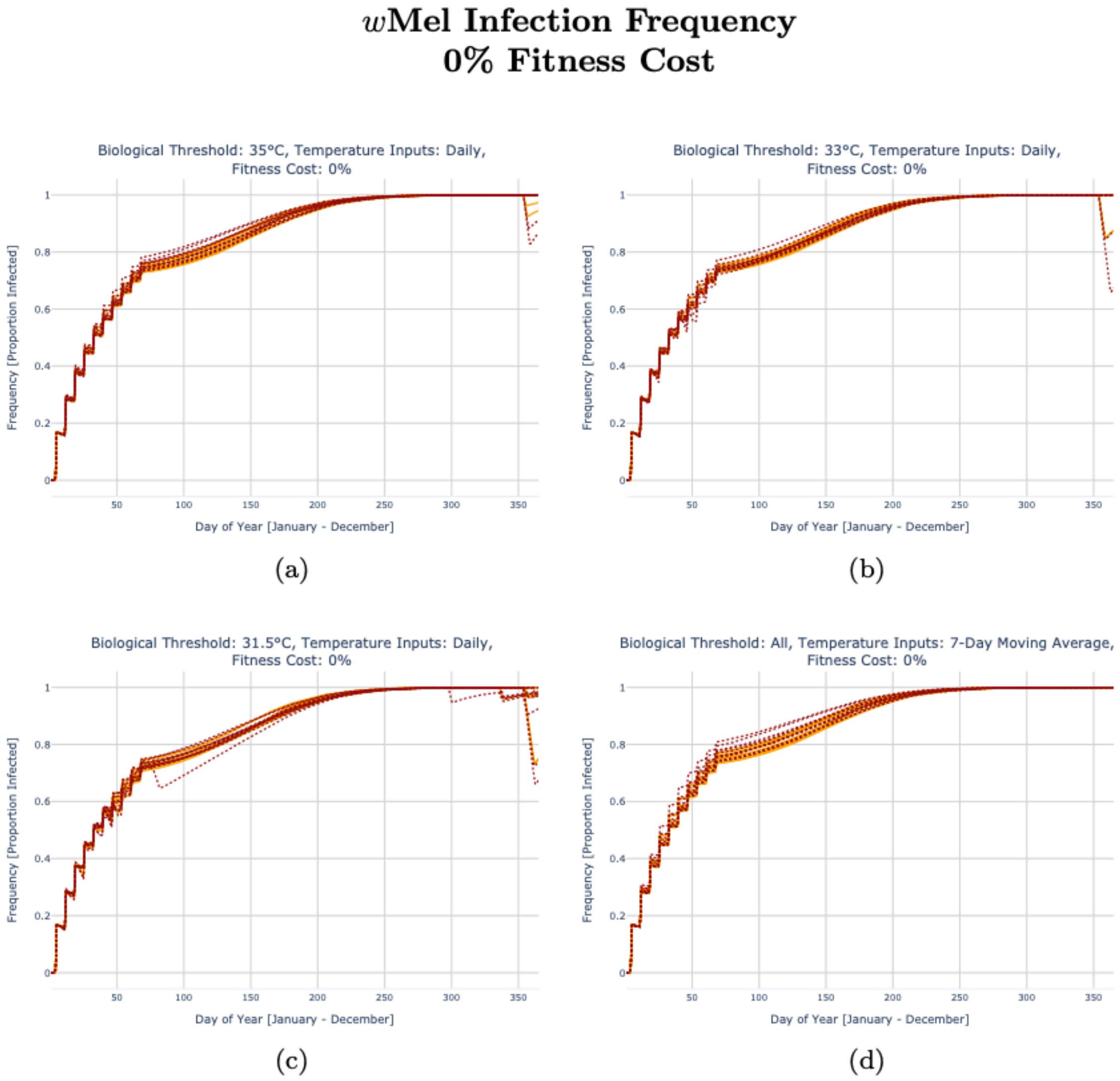

Supplementary Fig. 7: Effect of future heatwaves on *w*Mel infection frequency under Cairns 2030 & 2050 climate scenarios, assuming 0% fitness cost.

Population Dynamics and wMel Infection Frequency in Future Years for Alternative Biological Thresholds: Region of Nha Trang City, Vietnam

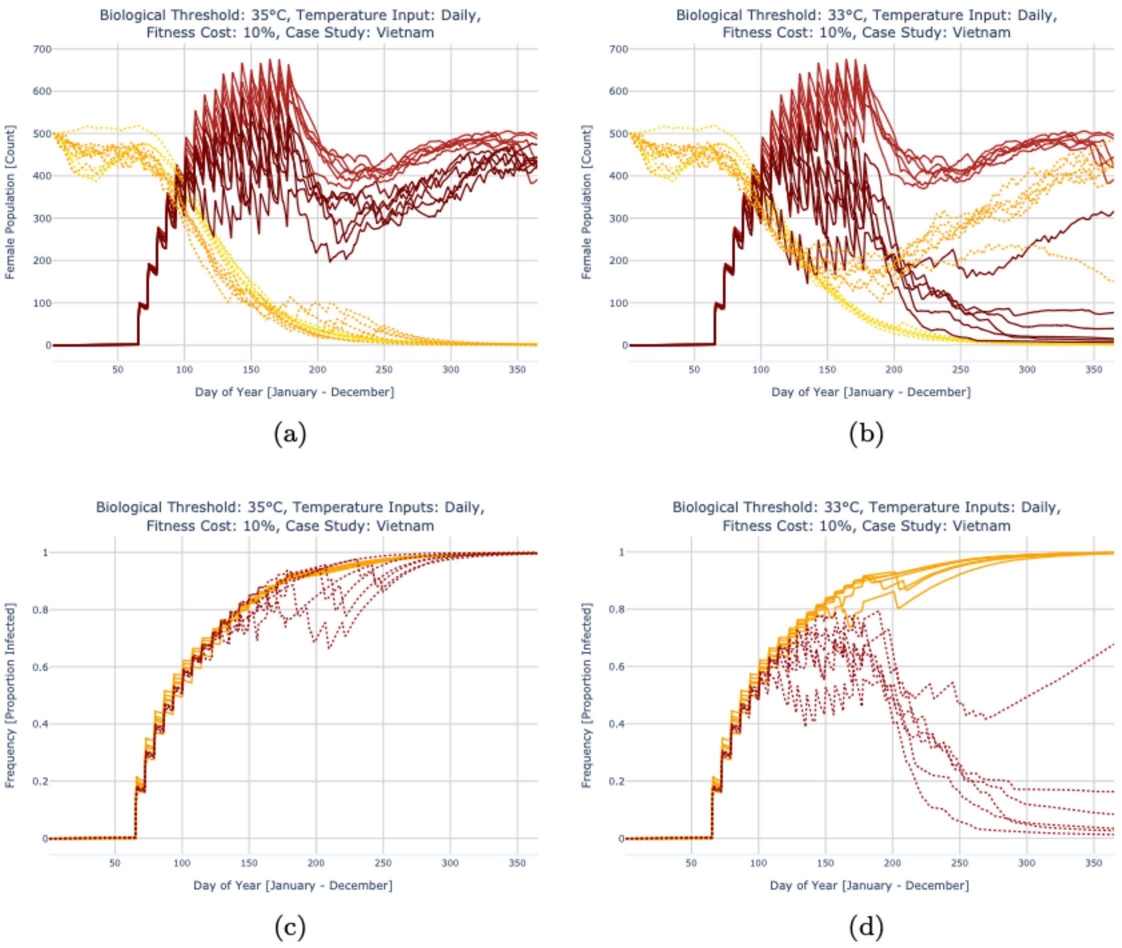

Supplementary Fig. 8: Population dynamics and wMel infection frequency as simulated for the Nha Trang City region of Vietnam. Panels (a) & (b) show replacement and suppression dynamics for future heatwave years and their baselines, assuming 35.0°C and 33.0°C thermal thresholds respectively. Panels (c) & (d) display the effect of future heatwaves on the frequency of wMel infection in an adult female *Ae. aegypti* population for those same thresholds. All panels presume a 10% fitness cost.

Table 1: **Historical: Biological Threshold 35.0°C & Fitness Cost 10%**

**Location**	**Temperature**	**RES**	**Year**	**Scenario**
Cairns	25.08	81.33590834019788	1990	historical
Cairns	24.86	82.39726798358345	1991	historical
Cairns	25.23	82.28447513209416	1992	historical
Cairns	24.83	82.82740504193607	1993	historical
Cairns	24.84	82.69757681492975	1994	historical
Cairns	25.39	81.63957921383597	1995	historical
Cairns	25.12	81.26707132795767	1996	historical
Cairns	24.35	82.75956820992751	1997	historical
Cairns	25.85	81.74554113074079	1998	historical
Cairns	24.84	82.10489236400016	1999	historical
Cairns	24.72	81.9754511071369	2000	historical
Cairns	25.1	82.73593648454326	2001	historical
Cairns	25.23	81.50598162995483	2002	historical
Cairns	25.25	81.85886865958705	2003	historical
Cairns	25.01	81.92080922006643	2004	historical
Cairns	25.41	81.95487911127039	2005	historical
Cairns	24.99	81.19012675592695	2006	historical
Cairns	25.03	81.5942299549805	2007	historical
Cairns	25.25	82.26740223703378	2008	historical
Cairns	25.23	81.52975594523866	2009	historical
Cairns	26.04	81.52287752920083	2010	historical
Cairns	24.84	81.44802360787699	2011	historical
Cairns	25.02	81.72104240144049	2012	historical
Cairns	25.31	82.0471848204369	2013	historical
Cairns	25.06	82.40480723261139	2014	historical
Cairns	25.32	81.96672403685263	2015	historical
Cairns	26.08	81.1242913090956	2016	historical
Cairns	25.72	81.29082713384813	2017	historical
Cairns	25.41	82.04395526842842	2018	historical
Cairns	25.22	81.8430523014845	2019	historical

Table 2: **2030s: Biological Threshold 35.0°C & Fitness Cost 10%**

**Location**	**Temperature**	**RES**	**Year**	**Scenario**
Cairns	25.87	81.03509133411109	2024	RCP8.5
Cairns	25.79	81.03766376568893	2024	RCP4.5
Cairns	25.65	81.8157774682337	2025	RCP8.5
Cairns	25.58	81.85432390594521	2025	RCP4.5
Cairns	26.02	82.3642868187033	2026	RCP8.5
Cairns	25.94	82.3523707206844	2026	RCP4.5
Cairns	25.55	81.70346763142928	2027	RCP4.5
Cairns	25.62	81.6195701835356	2027	RCP8.5
Cairns	25.56	82.83515708686993	2028	RCP4.5
Cairns	25.63	82.85214124797673	2028	RCP8.5
Cairns	26.1	81.36153194424544	2029	RCP4.5
Cairns	26.18	81.36281948269215	2029	RCP8.5
Cairns	25.91	81.11553827603856	2030	RCP8.5
Cairns	25.84	81.10787469686375	2030	RCP4.5
Cairns	25.06	81.73292338138394	2031	RCP4.5
Cairns	25.14	81.65981853152303	2031	RCP8.5
Cairns	26.64	81.4296078109626	2032	RCP8.5
Cairns	26.57	81.43680248329423	2032	RCP4.5
Cairns	25.63	81.38688337786101	2033	RCP8.5
Cairns	25.55	81.42822437305198	2033	RCP4.5
Cairns	25.51	80.96210369520742	2034	RCP8.5
Cairns	25.44	81.03183084947906	2034	RCP4.5
Cairns	25.82	81.59477240198072	2035	RCP4.5
Cairns	25.89	81.5185042150609	2035	RCP8.5
Cairns	26.02	81.84464616317214	2036	RCP8.5
Cairns	25.95	81.80953527422976	2036	RCP4.5
Cairns	26.04	81.24287092937965	2037	RCP8.5
Cairns	25.97	81.27475070785961	2037	RCP4.5
Cairns	25.8	81.59472525521613	2038	RCP8.5
Cairns	25.73	81.6199739103452	2038	RCP4.5
Cairns	26.13	81.61262303296594	2039	RCP4.5
Cairns	26.2	81.60442516044336	2039	RCP8.5

Table 3: **2050s: Biological Threshold 35.0°C & Fitness Cost 10%**

Location	Temperature	RES	Year	Scenario
Cairns	26.55	81.55688235107908	2044	RCP8.5
Cairns	26.2	81.18346623226095	2044	RCP4.5
Cairns	25.98	81.82047030062888	2045	RCP4.5
Cairns	26.33	81.90646086875331	2045	RCP8.5
Cairns	26.35	82.58401029436759	2046	RCP4.5
Cairns	26.7	82.94475162415564	2046	RCP8.5
Cairns	25.95	81.39273869997797	2047	RCP4.5
Cairns	26.3	81.21236541091864	2047	RCP8.5
Cairns	25.96	83.05553867520707	2048	RCP4.5
Cairns	26.31	83.3816364538241	2048	RCP8.5
Cairns	26.86	81.6950239814207	2049	RCP8.5
Cairns	26.51	81.44814960759732	2049	RCP4.5
Cairns	26.24	81.30653980496166	2050	RCP4.5
Cairns	26.59	81.73245529012478	2050	RCP8.5
Cairns	25.82	81.34352185214941	2051	RCP8.5
Cairns	25.47	81.48701754232606	2051	RCP4.5
Cairns	27.33	81.88680897988316	2052	RCP8.5
Cairns	26.98	81.53802289121232	2052	RCP4.5
Cairns	26.31	81.47349623323471	2053	RCP8.5
Cairns	25.96	81.36559442072375	2053	RCP4.5
Cairns	26.2	80.90457064642048	2054	RCP8.5
Cairns	25.85	80.89176815246839	2054	RCP4.5
Cairns	26.22	81.29154558429036	2055	RCP4.5
Cairns	26.57	81.17598515483849	2055	RCP8.5
Cairns	26.71	82.66060905629399	2056	RCP8.5
Cairns	26.36	82.1536112762426	2056	RCP4.5
Cairns	26.72	81.41511122466248	2057	RCP8.5
Cairns	26.37	81.24752986870976	2057	RCP4.5
Cairns	26.49	81.97779444570733	2058	RCP8.5
Cairns	26.14	81.71516674209441	2058	RCP4.5
Cairns	26.89	81.93203382317314	2059	RCP8.5
Cairns	26.54	81.67851185302061	2059	RCP4.5

Table 4: **Historical: Biological Threshold 35.0°C & Fitness Cost 0%**

Location	Temperature	RES	Year	Scenario
Cairns	25.08	82.7101405849748	1990	historical
Cairns	24.86	83.65158112642986	1991	historical
Cairns	25.23	83.56247545652158	1992	historical
Cairns	24.83	84.01016112459466	1993	historical
Cairns	24.84	83.93678617463623	1994	historical
Cairns	25.39	82.97584614212901	1995	historical
Cairns	25.12	82.638060037118	1996	historical
Cairns	24.35	83.97540454938557	1997	historical
Cairns	25.85	83.05979858469544	1998	historical
Cairns	24.84	83.38436840758426	1999	historical
Cairns	24.72	83.28826311187073	2000	historical
Cairns	25.1	83.944174030547	2001	historical
Cairns	25.23	82.86675217451238	2002	historical
Cairns	25.25	83.15133616502173	2003	historical
Cairns	25.01	83.22733128283838	2004	historical
Cairns	25.41	83.24346700888019	2005	historical
Cairns	24.99	82.55764725404596	2006	historical
Cairns	25.03	82.94573743852062	2007	historical
Cairns	25.25	83.54427222126715	2008	historical
Cairns	25.23	82.85839815345739	2009	historical
Cairns	26.04	82.8477227097823	2010	historical
Cairns	24.84	82.82069101821834	2011	historical
Cairns	25.02	83.06957885696156	2012	historical
Cairns	25.31	83.33816876091814	2013	historical
Cairns	25.06	83.65244731409244	2014	historical
Cairns	25.32	83.2554773037558	2015	historical
Cairns	26.08	82.50282577484428	2016	historical
Cairns	25.72	82.64112147201782	2017	historical
Cairns	25.41	83.32190254764991	2018	historical
Cairns	25.22	83.15790802934757	2019	historical

Table 5: **2030s: Biological Threshold 35.0°C & Fitness Cost 0%**

Location	Temperature	RES	Year	Scenario
Cairns	25.79	82.45175411826688	2024	RCP4.5
Cairns	25.87	82.45480139099706	2024	RCP8.5
Cairns	25.58	83.15619467958794	2025	RCP4.5
Cairns	25.65	83.12267321735852	2025	RCP8.5
Cairns	25.94	83.63892499877281	2026	RCP4.5
Cairns	26.02	83.65081626802481	2026	RCP8.5
Cairns	25.62	82.93400289242966	2027	RCP8.5
Cairns	25.55	83.00368742423463	2027	RCP4.5
Cairns	25.63	84.09395590253713	2028	RCP8.5
Cairns	25.56	84.07469631976852	2028	RCP4.5
Cairns	26.1	82.74018616240805	2029	RCP4.5
Cairns	26.18	82.74062022577428	2029	RCP8.5
Cairns	25.84	82.49780932804205	2030	RCP4.5
Cairns	25.91	82.50620704873471	2030	RCP8.5
Cairns	25.06	83.04902416207936	2031	RCP4.5
Cairns	25.14	82.98331057940855	2031	RCP8.5
Cairns	26.57	82.79730588228061	2032	RCP4.5
Cairns	26.64	82.79690775380183	2032	RCP8.5
Cairns	25.55	82.78562764363609	2033	RCP4.5
Cairns	25.63	82.75008685188592	2033	RCP8.5
Cairns	25.44	82.43538592651373	2034	RCP4.5
Cairns	25.51	82.37479244841626	2034	RCP8.5
Cairns	25.82	82.9209462645236	2035	RCP4.5
Cairns	25.89	82.85082667238451	2035	RCP8.5
Cairns	25.95	83.14860668642831	2036	RCP4.5
Cairns	26.02	83.18328748031063	2036	RCP8.5
Cairns	25.97	82.6322052138121	2037	RCP4.5
Cairns	26.04	82.6043033609292	2037	RCP8.5
Cairns	25.8	82.9486809498261	2038	RCP8.5
Cairns	25.73	82.96910185049015	2038	RCP4.5
Cairns	26.13	82.94612165324602	2039	RCP4.5
Cairns	26.2	82.93843892713795	2039	RCP8.5

Table 6: **2050s: Biological Threshold 35.0°C & Fitness Cost 0%**

Location	Temperature	RES	Year	Scenario
Cairns	26.2	82.5966650906967	2044	RCP4.5
Cairns	26.55	82.95799908407626	2044	RCP8.5
Cairns	26.33	83.21838200963371	2045	RCP8.5
Cairns	25.98	83.12715053715209	2045	RCP4.5
Cairns	26.7	84.20603760623321	2046	RCP8.5
Cairns	26.35	83.85718702781767	2046	RCP4.5
Cairns	25.95	82.73351192258808	2047	RCP4.5
Cairns	26.3	82.59484726429756	2047	RCP8.5
Cairns	25.96	84.28430333643342	2048	RCP4.5
Cairns	26.31	84.60328488700863	2048	RCP8.5
Cairns	26.86	83.0717446020896	2049	RCP8.5
Cairns	26.51	82.82473545720362	2049	RCP4.5
Cairns	26.59	83.08675457217262	2050	RCP8.5
Cairns	26.24	82.68484581223163	2050	RCP4.5
Cairns	25.47	82.82651418742996	2051	RCP4.5
Cairns	25.82	82.70184801058365	2051	RCP8.5
Cairns	27.33	83.2467193127129	2052	RCP8.5
Cairns	26.98	82.90636357214635	2052	RCP4.5
Cairns	26.31	82.85143298528293	2053	RCP8.5
Cairns	25.96	82.73481198336172	2053	RCP4.5
Cairns	26.2	82.33044561511788	2054	RCP8.5
Cairns	25.85	82.30717379607124	2054	RCP4.5
Cairns	26.22	82.65069296510336	2055	RCP4.5
Cairns	26.57	82.56635638474366	2055	RCP8.5
Cairns	26.36	83.47088266952163	2056	RCP4.5
Cairns	26.71	83.9495503640731	2056	RCP8.5
Cairns	26.72	82.78488439327026	2057	RCP8.5
Cairns	26.37	82.61289976171491	2057	RCP4.5
Cairns	26.49	83.31947301628631	2058	RCP8.5
Cairns	26.14	83.06422075591587	2058	RCP4.5
Cairns	26.89	83.26495527570734	2059	RCP8.5
Cairns	26.54	83.01172388900761	2059	RCP4.5

Table 7: **Historical: Biological Threshold 35.0°C & Fitness Cost 20%**

Location	Temperature	RES	Year	Scenario
Cairns	25.08	79.6964778412146	1990	historical
Cairns	24.86	80.91787513454216	1991	historical
Cairns	25.23	80.77311190064307	1992	historical
Cairns	24.83	81.44492817299772	1993	historical
Cairns	24.84	81.2415597968298	1994	historical
Cairns	25.39	80.05766837378997	1995	historical
Cairns	25.12	79.64351040816591	1996	historical
Cairns	24.35	81.31423912492033	1997	historical
Cairns	25.85	80.20263001738579	1998	historical
Cairns	24.84	80.58914450558571	1999	historical
Cairns	24.72	80.42872030440174	2000	historical
Cairns	25.1	81.32599407061139	2001	historical
Cairns	25.23	79.88937970252486	2002	historical
Cairns	25.25	80.32849597316681	2003	historical
Cairns	25.01	80.36741719589581	2004	historical
Cairns	25.41	80.42744465461558	2005	historical
Cairns	24.99	79.57018787650051	2006	historical
Cairns	25.03	79.98469581057907	2007	historical
Cairns	25.25	80.74749849067321	2008	historical
Cairns	25.23	79.96152725744776	2009	historical
Cairns	26.04	79.96018772070266	2010	historical
Cairns	24.84	79.81881820561273	2011	historical
Cairns	25.02	80.11498939569645	2012	historical
Cairns	25.31	80.52568739307301	2013	historical
Cairns	25.06	80.94472463314628	2014	historical
Cairns	25.32	80.4435805860594	2015	historical
Cairns	26.08	79.50751158136318	2016	historical
Cairns	25.72	79.6819134296295	2017	historical
Cairns	25.41	80.54360404384218	2018	historical
Cairns	25.22	80.29109205313787	2019	historical

Table 8: **2030s: Biological Threshold 35.0°C & Fitness Cost 20%**

**Location**	**Temperature**	**RES**	**Year**	**Scenario**
Cairns	25.79	79.36035623762567	2024	RCP4.5
Cairns	25.87	79.35565568425201	2024	RCP8.5
Cairns	25.58	80.32110811577581	2025	RCP4.5
Cairns	25.65	80.28142656854163	2025	RCP8.5
Cairns	25.94	80.84916136286469	2026	RCP4.5
Cairns	26.02	80.86188180205386	2026	RCP8.5
Cairns	25.55	80.16357048557316	2027	RCP4.5
Cairns	25.62	80.07063121594612	2027	RCP8.5
Cairns	25.63	81.40725924137062	2028	RCP8.5
Cairns	25.56	81.38975796472138	2028	RCP4.5
Cairns	26.1	79.73863055206414	2029	RCP4.5
Cairns	26.18	79.73469170347767	2029	RCP8.5
Cairns	25.91	79.4812992934887	2030	RCP8.5
Cairns	25.84	79.47631622566374	2030	RCP4.5
Cairns	25.06	80.16653269130803	2031	RCP4.5
Cairns	25.14	80.08413421896002	2031	RCP8.5
Cairns	26.57	79.84252511823514	2032	RCP4.5
Cairns	26.64	79.83151383219206	2032	RCP8.5
Cairns	25.63	79.77860708420216	2033	RCP8.5
Cairns	25.55	79.82618883037887	2033	RCP4.5
Cairns	25.51	79.29611607228058	2034	RCP8.5
Cairns	25.44	79.37138252223203	2034	RCP4.5
Cairns	25.82	80.03642516639141	2035	RCP4.5
Cairns	25.89	79.94771873670952	2035	RCP8.5
Cairns	26.02	80.27812734991961	2036	RCP8.5
Cairns	25.95	80.23889424169005	2036	RCP4.5
Cairns	26.04	79.63431252312223	2037	RCP8.5
Cairns	25.97	79.6705666716735	2037	RCP4.5
Cairns	25.8	80.00388640998186	2038	RCP8.5
Cairns	25.73	80.03750511521255	2038	RCP4.5
Cairns	26.2	80.03376850974507	2039	RCP8.5
Cairns	26.13	80.0479330041884	2039	RCP4.5

Table 9: **2050s: Biological Threshold 35.0°C & Fitness Cost 20%**

**Location**	**Temperature**	**RES**	**Year**	**Scenario**
Cairns	26.55	79.91386696491169	2044	RCP8.5
Cairns	26.2	79.51803648378505	2044	RCP4.5
Cairns	25.98	80.28471675169118	2045	RCP4.5
Cairns	26.33	80.37352813010204	2045	RCP8.5
Cairns	26.35	81.10372620258869	2046	RCP4.5
Cairns	26.7	81.48472481381332	2046	RCP8.5
Cairns	25.95	79.80577794141752	2047	RCP4.5
Cairns	26.3	79.57611118838389	2047	RCP8.5
Cairns	26.31	81.97148258054621	2048	RCP8.5
Cairns	25.96	81.62794891989232	2048	RCP4.5
Cairns	26.86	80.08397974703011	2049	RCP8.5
Cairns	26.51	79.82797316998825	2049	RCP4.5
Cairns	26.59	80.14585554629117	2050	RCP8.5
Cairns	26.24	79.69255720862563	2050	RCP4.5
Cairns	25.47	79.90043975830595	2051	RCP4.5
Cairns	25.82	79.72967267854044	2051	RCP8.5
Cairns	27.33	80.30003070625233	2052	RCP8.5
Cairns	26.98	79.93956626233526	2052	RCP4.5
Cairns	25.96	79.75215048728576	2053	RCP4.5
Cairns	26.31	79.85689598198398	2053	RCP8.5
Cairns	26.2	79.2269960524467	2054	RCP8.5
Cairns	25.85	79.2230771376367	2054	RCP4.5
Cairns	26.57	79.54407041331852	2055	RCP8.5
Cairns	26.22	79.68474377281119	2055	RCP4.5
Cairns	26.36	80.61575892868717	2056	RCP4.5
Cairns	26.71	81.16118263573985	2056	RCP8.5
Cairns	26.72	79.8023048954046	2057	RCP8.5
Cairns	26.37	79.63285676555839	2057	RCP4.5
Cairns	26.14	80.13969900316096	2058	RCP4.5
Cairns	26.49	80.41738131021987	2058	RCP8.5
Cairns	26.89	80.3654343455225	2059	RCP8.5
Cairns	26.54	80.1064796234497	2059	RCP4.5

Table 10: **Historical: Biological Threshold 33.0°C & Fitness Cost 10%**

**Location**	**Temperature**	**RES**	**Year**	**Scenario**
Cairns	25.08	81.33590834019788	1990	historical
Cairns	24.86	82.39726808019789	1991	historical
Cairns	25.23	82.28447513209416	1992	historical
Cairns	24.83	82.82740504193607	1993	historical
Cairns	24.84	82.44719543867173	1994	historical
Cairns	25.39	81.53001709924061	1995	historical
Cairns	25.12	81.26707132795767	1996	historical
Cairns	24.35	82.75956820992751	1997	historical
Cairns	25.85	81.74554113074079	1998	historical
Cairns	24.84	82.10489236400016	1999	historical
Cairns	24.72	81.9754511071369	2000	historical
Cairns	25.1	82.73593648454326	2001	historical
Cairns	25.23	81.50598162995483	2002	historical
Cairns	25.25	81.85886865958705	2003	historical
Cairns	25.01	81.92080922006643	2004	historical
Cairns	25.41	81.95487911127039	2005	historical
Cairns	24.99	81.19012675592695	2006	historical
Cairns	25.03	81.5942299549805	2007	historical
Cairns	25.25	82.26740223703378	2008	historical
Cairns	25.23	81.52975594523866	2009	historical
Cairns	26.04	81.52287752920083	2010	historical
Cairns	24.84	81.44802360787699	2011	historical
Cairns	25.02	81.72104240144049	2012	historical
Cairns	25.31	82.0471848204369	2013	historical
Cairns	25.06	82.40480723261139	2014	historical
Cairns	25.32	81.96672403685263	2015	historical
Cairns	26.08	81.1242913090956	2016	historical
Cairns	25.72	81.29082713384813	2017	historical
Cairns	25.41	81.35097386488542	2018	historical
Cairns	25.22	81.54558851539264	2019	historical

Table 11: **2030s: Biological Threshold 33.0°C & Fitness Cost 10%**

Location	Temperature	RES	Year	Scenario
Cairns	25.87	81.03509133411109	2024	RCP8.5
Cairns	25.79	81.03766376568893	2024	RCP4.5
Cairns	25.65	81.6066809539903	2025	RCP8.5
Cairns	25.58	81.64339783592143	2025	RCP4.5
Cairns	26.02	82.15217777525204	2026	RCP8.5
Cairns	25.94	82.3523707206844	2026	RCP4.5
Cairns	25.55	81.70346763142928	2027	RCP4.5
Cairns	25.62	81.6195701835356	2027	RCP8.5
Cairns	25.56	82.60209294550923	2028	RCP4.5
Cairns	25.63	82.56479437229561	2028	RCP8.5
Cairns	26.18	81.23005801961585	2029	RCP8.5
Cairns	26.1	81.22915966747964	2029	RCP4.5
Cairns	25.84	81.10787469686375	2030	RCP4.5
Cairns	25.91	81.11553827603856	2030	RCP8.5
Cairns	25.14	81.65981853152303	2031	RCP8.5
Cairns	25.06	81.73292338138394	2031	RCP4.5
Cairns	26.57	81.43680248329423	2032	RCP4.5
Cairns	26.64	81.4296078109626	2032	RCP8.5
Cairns	25.63	81.38688337786101	2033	RCP8.5
Cairns	25.55	81.42822437305198	2033	RCP4.5
Cairns	25.44	81.03183084947906	2034	RCP4.5
Cairns	25.51	80.96210369520742	2034	RCP8.5
Cairns	25.89	81.5185042150609	2035	RCP8.5
Cairns	25.82	81.59477240198072	2035	RCP4.5
Cairns	26.02	81.84464616317214	2036	RCP8.5
Cairns	25.95	81.80953527422976	2036	RCP4.5
Cairns	25.97	81.27475070785961	2037	RCP4.5
Cairns	26.04	81.24287092937965	2037	RCP8.5
Cairns	25.73	81.6199739103452	2038	RCP4.5
Cairns	25.8	81.59472525521613	2038	RCP8.5
Cairns	26.13	81.61262303296594	2039	RCP4.5
Cairns	26.2	81.60442516044336	2039	RCP8.5

Table 12: **2050s: Biological Threshold 33.0°C & Fitness Cost 10%**

Location	Temperature	RES	Year	Scenario
Cairns	26.55	81.55688235107908	2044	RCP8.5
Cairns	26.2	81.18346623226095	2044	RCP4.5
Cairns	25.98	81.61574904914829	2045	RCP4.5
Cairns	26.33	81.70411250495808	2045	RCP8.5
Cairns	26.7	82.14824978558892	2046	RCP8.5
Cairns	26.35	82.37073338968092	2046	RCP4.5
Cairns	25.95	81.39273869997797	2047	RCP4.5
Cairns	26.3	81.21236541091864	2047	RCP8.5
Cairns	26.31	83.05403195395893	2048	RCP8.5
Cairns	25.96	82.78417707789883	2048	RCP4.5
Cairns	26.86	81.68209244743994	2049	RCP8.5
Cairns	26.51	81.35893208369151	2049	RCP4.5
Cairns	26.24	81.30653980496166	2050	RCP4.5
Cairns	26.59	81.73245529012478	2050	RCP8.5
Cairns	25.47	81.48701754232606	2051	RCP4.5
Cairns	25.82	81.34352185214941	2051	RCP8.5
Cairns	26.98	81.53802289121232	2052	RCP4.5
Cairns	27.33	81.88680897988316	2052	RCP8.5
Cairns	26.31	81.47349623323471	2053	RCP8.5
Cairns	25.96	81.36559442072375	2053	RCP4.5
Cairns	25.85	80.89176815246839	2054	RCP4.5
Cairns	26.2	80.90457064642048	2054	RCP8.5
Cairns	26.57	81.17598515483849	2055	RCP8.5
Cairns	26.22	81.29154558429036	2055	RCP4.5
Cairns	26.71	82.66060905629399	2056	RCP8.5
Cairns	26.36	82.1536112762426	2056	RCP4.5
Cairns	26.72	81.41511122466248	2057	RCP8.5
Cairns	26.37	81.24752986870976	2057	RCP4.5
Cairns	26.14	81.71516674209441	2058	RCP4.5
Cairns	26.49	81.97779444570733	2058	RCP8.5
Cairns	26.54	81.67851185302061	2059	RCP4.5
Cairns	26.89	81.93203382317314	2059	RCP8.5

Table 13: **Historical: Biological Threshold 33.0°C & Fitness Cost 0%**

**Location**	**Temperature**	**RES**	**Year**	**Scenario**
Cairns	25.08	82.7101405849748	1990	historical
Cairns	24.86	83.65158123142896	1991	historical
Cairns	25.23	83.56247545652158	1992	historical
Cairns	24.83	84.01016112459466	1993	historical
Cairns	24.84	83.70810382354732	1994	historical
Cairns	25.39	82.87337895866072	1995	historical
Cairns	25.12	82.638060037118	1996	historical
Cairns	24.35	83.97540454938557	1997	historical
Cairns	25.85	83.05979858469544	1998	historical
Cairns	24.84	83.38436840758426	1999	historical
Cairns	24.72	83.28826311187073	2000	historical
Cairns	25.1	83.944174030547	2001	historical
Cairns	25.23	82.86675217451238	2002	historical
Cairns	25.25	83.15133616502173	2003	historical
Cairns	25.01	83.22733128283838	2004	historical
Cairns	25.41	83.24346700888019	2005	historical
Cairns	24.99	82.55764725404596	2006	historical
Cairns	25.03	82.94573743852062	2007	historical
Cairns	25.25	83.54427222126715	2008	historical
Cairns	25.23	82.85839815345739	2009	historical
Cairns	26.04	82.8477227097823	2010	historical
Cairns	24.84	82.82069101821834	2011	historical
Cairns	25.02	83.06957885696156	2012	historical
Cairns	25.31	83.33816876091814	2013	historical
Cairns	25.06	83.65244731409244	2014	historical
Cairns	25.32	83.2554773037558	2015	historical
Cairns	26.08	82.50282577484428	2016	historical
Cairns	25.72	82.64112147201782	2017	historical
Cairns	25.41	82.68759604532015	2018	historical
Cairns	25.22	82.8971445438679	2019	historical

Table 14: **2030s: Biological Threshold 33.0°C & Fitness Cost 0%**

**Location**	**Temperature**	**RES**	**Year**	**Scenario**
Cairns	25.87	82.45480139099706	2024	RCP8.5
Cairns	25.79	82.45175411826688	2024	RCP4.5
Cairns	25.58	82.96063075781612	2025	RCP4.5
Cairns	25.65	82.92913149107211	2025	RCP8.5
Cairns	25.94	83.63892499877281	2026	RCP4.5
Cairns	26.02	83.4639775939346	2026	RCP8.5
Cairns	25.62	82.93400289242966	2027	RCP8.5
Cairns	25.55	83.00368742423463	2027	RCP4.5
Cairns	25.63	83.82847028941967	2028	RCP8.5
Cairns	25.56	83.86061687816338	2028	RCP4.5
Cairns	26.18	82.6147996395714	2029	RCP8.5
Cairns	26.1	82.61753278290867	2029	RCP4.5
Cairns	25.84	82.49780932804205	2030	RCP4.5
Cairns	25.91	82.50620704873471	2030	RCP8.5
Cairns	25.14	82.98331057940855	2031	RCP8.5
Cairns	25.06	83.04902416207936	2031	RCP4.5
Cairns	26.64	82.79690775380183	2032	RCP8.5
Cairns	26.57	82.79730588228061	2032	RCP4.5
Cairns	25.63	82.75008685188592	2033	RCP8.5
Cairns	25.55	82.78562764363609	2033	RCP4.5
Cairns	25.51	82.37479244841626	2034	RCP8.5
Cairns	25.44	82.43538592651373	2034	RCP4.5
Cairns	25.82	82.9209462645236	2035	RCP4.5
Cairns	25.89	82.85082667238451	2035	RCP8.5
Cairns	25.95	83.14860668642831	2036	RCP4.5
Cairns	26.02	83.18328748031063	2036	RCP8.5
Cairns	26.04	82.6043033609292	2037	RCP8.5
Cairns	25.97	82.6322052138121	2037	RCP4.5
Cairns	25.73	82.96910185049015	2038	RCP4.5
Cairns	25.8	82.9486809498261	2038	RCP8.5
Cairns	26.2	82.93843892713795	2039	RCP8.5
Cairns	26.13	82.94612165324602	2039	RCP4.5

Table 15: **2050s: Biological Threshold 33.0°C & Fitness Cost 0%**

Location	Temperature	RES	Year	Scenario
Cairns	26.55	82.95799908407626	2044	RCP8.5
Cairns	26.2	82.5966650906967	2044	RCP4.5
Cairns	25.98	82.93868894277621	2045	RCP4.5
Cairns	26.33	83.03194671392541	2045	RCP8.5
Cairns	26.7	83.49571260556294	2046	RCP8.5
Cairns	26.35	83.66652278724816	2046	RCP4.5
Cairns	25.95	82.73351192258808	2047	RCP4.5
Cairns	26.3	82.59484726429756	2047	RCP8.5
Cairns	26.31	84.29830587215909	2048	RCP8.5
Cairns	25.96	84.03647427058542	2048	RCP4.5
Cairns	26.86	83.05773015167773	2049	RCP8.5
Cairns	26.51	82.74009558339547	2049	RCP4.5
Cairns	26.59	83.08675457217262	2050	RCP8.5
Cairns	26.24	82.68484581223163	2050	RCP4.5
Cairns	25.47	82.82651418742996	2051	RCP4.5
Cairns	25.82	82.70184801058365	2051	RCP8.5
Cairns	26.98	82.90636357214635	2052	RCP4.5
Cairns	27.33	83.2467193127129	2052	RCP8.5
Cairns	25.96	82.73481198336172	2053	RCP4.5
Cairns	26.31	82.85143298528293	2053	RCP8.5
Cairns	26.2	82.33044561511788	2054	RCP8.5
Cairns	25.85	82.30717379607124	2054	RCP4.5
Cairns	26.22	82.65069296510336	2055	RCP4.5
Cairns	26.57	82.56635638474366	2055	RCP8.5
Cairns	26.36	83.47088266952163	2056	RCP4.5
Cairns	26.71	83.9495503640731	2056	RCP8.5
Cairns	26.72	82.78488439327026	2057	RCP8.5
Cairns	26.37	82.61289976171491	2057	RCP4.5
Cairns	26.14	83.06422075591587	2058	RCP4.5
Cairns	26.49	83.31947301628631	2058	RCP8.5
Cairns	26.54	83.01172388900761	2059	RCP4.5
Cairns	26.89	83.26495527570734	2059	RCP8.5

Table 16: **Historical: Biological Threshold 33.0°C & Fitness Cost 20%**

Location	Temperature	RES	Year	Scenario
Cairns	25.08	79.6964778412146	1990	historical
Cairns	24.86	80.91787525305844	1991	historical
Cairns	25.23	80.77311190064307	1992	historical
Cairns	24.83	81.44492817299772	1993	historical
Cairns	24.84	80.96304993341224	1994	historical
Cairns	25.39	79.93985220095958	1995	historical
Cairns	25.12	79.64351040816591	1996	historical
Cairns	24.35	81.31423912492033	1997	historical
Cairns	25.85	80.20263001738579	1998	historical
Cairns	24.84	80.58914450558571	1999	historical
Cairns	24.72	80.42872030440174	2000	historical
Cairns	25.1	81.32599407061139	2001	historical
Cairns	25.23	79.88937970252486	2002	historical
Cairns	25.25	80.32849597316681	2003	historical
Cairns	25.01	80.36741719589581	2004	historical
Cairns	25.41	80.42744465461558	2005	historical
Cairns	24.99	79.57018787650051	2006	historical
Cairns	25.03	79.98469581057907	2007	historical
Cairns	25.25	80.74749849067321	2008	historical
Cairns	25.23	79.96152725744776	2009	historical
Cairns	26.04	79.96018772070266	2010	historical
Cairns	24.84	79.81881820561273	2011	historical
Cairns	25.02	80.11498939569645	2012	historical
Cairns	25.31	80.52568739307301	2013	historical
Cairns	25.06	80.94472463314628	2014	historical
Cairns	25.32	80.4435805860594	2015	historical
Cairns	26.08	79.50751158136318	2016	historical
Cairns	25.72	79.6819134296295	2017	historical
Cairns	25.41	79.79172521014489	2018	historical
Cairns	25.22	79.94712602808025	2019	historical

Table 17: **2030s: Biological Threshold 33.0°C & Fitness Cost 20%**

**Location**	**Temperature**	**RES**	**Year**	**Scenario**
Cairns	25.87	79.35565568425201	2024	RCP8.5
Cairns	25.79	79.36035623762567	2024	RCP4.5
Cairns	25.65	80.05688206316222	2025	RCP8.5
Cairns	25.58	80.09480315637661	2025	RCP4.5
Cairns	26.02	80.620283464369	2026	RCP8.5
Cairns	25.94	80.84916136286469	2026	RCP4.5
Cairns	25.62	80.07063121594612	2027	RCP8.5
Cairns	25.55	80.16357048557316	2027	RCP4.5
Cairns	25.63	81.092133705656	2028	RCP8.5
Cairns	25.56	81.13092687628445	2028	RCP4.5
Cairns	26.1	79.60159403500616	2029	RCP4.5
Cairns	26.18	79.59482215997954	2029	RCP8.5
Cairns	25.91	79.4812992934887	2030	RCP8.5
Cairns	25.84	79.47631622566374	2030	RCP4.5
Cairns	25.14	80.08413421896002	2031	RCP8.5
Cairns	25.06	80.16653269130803	2031	RCP4.5
Cairns	26.57	79.84252511823514	2032	RCP4.5
Cairns	26.64	79.83151383219206	2032	RCP8.5
Cairns	25.63	79.77860708420216	2033	RCP8.5
Cairns	25.55	79.82618883037887	2033	RCP4.5
Cairns	25.51	79.29611607228058	2034	RCP8.5
Cairns	25.44	79.37138252223203	2034	RCP4.5
Cairns	25.82	80.03642516639141	2035	RCP4.5
Cairns	25.89	79.94771873670952	2035	RCP8.5
Cairns	26.02	80.27812734991961	2036	RCP8.5
Cairns	25.95	80.23889424169005	2036	RCP4.5
Cairns	25.97	79.6705666716735	2037	RCP4.5
Cairns	26.04	79.63431252312223	2037	RCP8.5
Cairns	25.73	80.03750511521255	2038	RCP4.5
Cairns	25.8	80.00388640998186	2038	RCP8.5
Cairns	26.13	80.0479330041884	2039	RCP4.5
Cairns	26.2	80.03376850974507	2039	RCP8.5

Table 18: **2050s: Biological Threshold 33.0°C & Fitness Cost 20%**

Location	Temperature	RES	Year	Scenario
Cairns	26.55	79.91386696491169	2044	RCP8.5
Cairns	26.2	79.51803648378505	2044	RCP4.5
Cairns	25.98	80.06408726623928	2045	RCP4.5
Cairns	26.33	80.15364614218375	2045	RCP8.5
Cairns	26.35	80.8593096469502	2046	RCP4.5
Cairns	26.7	80.57836282871352	2046	RCP8.5
Cairns	26.3	79.57611118838389	2047	RCP8.5
Cairns	25.95	79.80577794141752	2047	RCP4.5
Cairns	25.96	81.3332648121155	2048	RCP4.5
Cairns	26.31	81.61560926916371	2048	RCP8.5
Cairns	26.51	79.7372292910327	2049	RCP4.5
Cairns	26.86	80.07261948182791	2049	RCP8.5
Cairns	26.24	79.69255720862563	2050	RCP4.5
Cairns	26.59	80.14585554629117	2050	RCP8.5
Cairns	25.47	79.90043975830595	2051	RCP4.5
Cairns	25.82	79.72967267854044	2051	RCP8.5
Cairns	26.98	79.93956626233526	2052	RCP4.5
Cairns	27.33	80.30003070625233	2052	RCP8.5
Cairns	26.31	79.85689598198398	2053	RCP8.5
Cairns	25.96	79.75215048728576	2053	RCP4.5
Cairns	26.2	79.2269960524467	2054	RCP8.5
Cairns	25.85	79.2230771376367	2054	RCP4.5
Cairns	26.57	79.54407041331852	2055	RCP8.5
Cairns	26.22	79.68474377281119	2055	RCP4.5
Cairns	26.36	80.61575892868717	2056	RCP4.5
Cairns	26.71	81.16118263573985	2056	RCP8.5
Cairns	26.37	79.63285676555839	2057	RCP4.5
Cairns	26.72	79.8023048954046	2057	RCP8.5
Cairns	26.14	80.13969900316096	2058	RCP4.5
Cairns	26.49	80.41738131021987	2058	RCP8.5
Cairns	26.89	80.3654343455225	2059	RCP8.5
Cairns	26.54	80.1064796234497	2059	RCP4.5

Table 19: **Historical: Biological Threshold 31.5°C & Fitness Cost 10%**

Location	Temperature	RES	Year	Scenario
Cairns	25.08	81.33590834019788	1990	historical
Cairns	24.86	82.17183065672388	1991	historical
Cairns	25.23	81.53548565928365	1992	historical
Cairns	24.83	82.82740504193607	1993	historical
Cairns	24.84	82.27591747765275	1994	historical
Cairns	25.39	81.53442683415373	1995	historical
Cairns	25.12	81.26707132795767	1996	historical
Cairns	24.35	82.75956820992751	1997	historical
Cairns	25.85	81.74554113074079	1998	historical
Cairns	24.84	82.10489236400016	1999	historical
Cairns	24.72	81.9754511071369	2000	historical
Cairns	25.1	82.73593648454326	2001	historical
Cairns	25.23	81.50598162995483	2002	historical
Cairns	25.25	81.85886865958705	2003	historical
Cairns	25.01	81.92080922006643	2004	historical
Cairns	25.41	81.95487911127039	2005	historical
Cairns	24.99	81.19012675592695	2006	historical
Cairns	25.03	81.5942299549805	2007	historical
Cairns	25.25	82.26740223703378	2008	historical
Cairns	25.23	81.52975594523866	2009	historical
Cairns	26.04	81.52287752920083	2010	historical
Cairns	24.84	81.44802360787699	2011	historical
Cairns	25.02	81.72104240144049	2012	historical
Cairns	25.31	81.69456725635521	2013	historical
Cairns	25.06	82.40480723261139	2014	historical
Cairns	25.32	81.96672403685263	2015	historical
Cairns	26.08	81.1242913090956	2016	historical
Cairns	25.72	80.72873510767693	2017	historical
Cairns	25.41	81.10443667955491	2018	historical
Cairns	25.22	80.87752982498783	2019	historical

Table 20: **2030s: Biological Threshold 31.5°C & Fitness Cost 10%**

Location	Temperature	RES	Year	Scenario
Cairns	25.79	80.83819908110938	2024	RCP4.5
Cairns	25.87	80.83672260079821	2024	RCP8.5
Cairns	25.65	81.6066809539903	2025	RCP8.5
Cairns	25.58	81.64339783592143	2025	RCP4.5
Cairns	25.94	81.07187065840131	2026	RCP4.5
Cairns	26.02	80.79416174110571	2026	RCP8.5
Cairns	25.62	81.6195701835356	2027	RCP8.5
Cairns	25.55	81.70346763142928	2027	RCP4.5
Cairns	25.56	82.25971173585486	2028	RCP4.5
Cairns	25.63	82.27766071692875	2028	RCP8.5
Cairns	26.18	81.23414309709197	2029	RCP8.5
Cairns	26.1	81.2335622383319	2029	RCP4.5
Cairns	25.84	81.03922597257707	2030	RCP4.5
Cairns	25.91	81.04718541492667	2030	RCP8.5
Cairns	25.14	81.65981853152303	2031	RCP8.5
Cairns	25.06	81.73292338138394	2031	RCP4.5
Cairns	26.57	81.26577795316716	2032	RCP4.5
Cairns	26.64	81.25803008148809	2032	RCP8.5
Cairns	25.55	81.42822437305198	2033	RCP4.5
Cairns	25.63	81.38688337786101	2033	RCP8.5
Cairns	25.51	80.96210369520742	2034	RCP8.5
Cairns	25.44	81.03183084947906	2034	RCP4.5
Cairns	25.89	81.46823864116037	2035	RCP8.5
Cairns	25.82	81.54472876437562	2035	RCP4.5
Cairns	26.02	80.93680943982936	2036	RCP8.5
Cairns	25.95	80.91019663090056	2036	RCP4.5
Cairns	25.97	81.27475070785961	2037	RCP4.5
Cairns	26.04	81.24287092937965	2037	RCP8.5
Cairns	25.73	81.6199739103452	2038	RCP4.5
Cairns	25.8	81.59472525521613	2038	RCP8.5
Cairns	26.13	81.34273765688145	2039	RCP4.5
Cairns	26.2	81.33299244894069	2039	RCP8.5

Table 21: **2050s: Biological Threshold 31.5°C & Fitness Cost 10%**

**Location**	**Temperature**	**RES**	**Year**	**Scenario**
Cairns	26.55	81.35937293136564	2044	RCP8.5
Cairns	26.2	80.98752508419487	2044	RCP4.5
Cairns	26.33	81.10929793750631	2045	RCP8.5
Cairns	25.98	81.37450586873567	2045	RCP4.5
Cairns	26.35	80.90676064241782	2046	RCP4.5
Cairns	26.7	81.24474575466172	2046	RCP8.5
Cairns	25.95	81.08155249234628	2047	RCP4.5
Cairns	26.3	80.1473956039348	2047	RCP8.5
Cairns	25.96	82.44672946204159	2048	RCP4.5
Cairns	26.31	82.30317664436669	2048	RCP8.5
Cairns	26.86	81.18054595146336	2049	RCP8.5
Cairns	26.51	81.36407108223491	2049	RCP4.5
Cairns	26.59	81.66757244372272	2050	RCP8.5
Cairns	26.24	81.23877277389344	2050	RCP4.5
Cairns	25.47	81.48701754232606	2051	RCP4.5
Cairns	25.82	81.34352185214941	2051	RCP8.5
Cairns	27.33	81.22999918639314	2052	RCP8.5
Cairns	26.98	81.36762546926565	2052	RCP4.5
Cairns	26.31	80.99946997152203	2053	RCP8.5
Cairns	25.96	81.10317472167861	2053	RCP4.5
Cairns	26.2	80.90457064642048	2054	RCP8.5
Cairns	25.85	80.89176815246839	2054	RCP4.5
Cairns	26.22	81.24036153825251	2055	RCP4.5
Cairns	26.57	81.00056641561547	2055	RCP8.5
Cairns	26.71	81.22779768742605	2056	RCP8.5
Cairns	26.36	80.94190419931341	2056	RCP4.5
Cairns	26.72	81.41511122466248	2057	RCP8.5
Cairns	26.37	81.24752986870976	2057	RCP4.5
Cairns	26.14	81.65139436483656	2058	RCP4.5
Cairns	26.49	81.8475124990777	2058	RCP8.5
Cairns	26.89	81.40084074011862	2059	RCP8.5
Cairns	26.54	81.40169477124459	2059	RCP4.5

Table 22: **Historical: Biological Threshold 31.5°C & Fitness Cost 0%**

**Location**	**Temperature**	**RES**	**Year**	**Scenario**
Cairns	25.08	82.7101405849748	1990	historical
Cairns	24.86	83.4429913714158	1991	historical
Cairns	25.23	82.89835405311209	1992	historical
Cairns	24.83	84.01016112459466	1993	historical
Cairns	24.84	83.54744940221443	1994	historical
Cairns	25.39	82.87234435931872	1995	historical
Cairns	25.12	82.638060037118	1996	historical
Cairns	24.35	83.97540454938557	1997	historical
Cairns	25.85	83.05979858469544	1998	historical
Cairns	24.84	83.38436840758426	1999	historical
Cairns	24.72	83.28826311187073	2000	historical
Cairns	25.1	83.944174030547	2001	historical
Cairns	25.23	82.86675217451238	2002	historical
Cairns	25.25	83.15133616502173	2003	historical
Cairns	25.01	83.22733128283838	2004	historical
Cairns	25.41	83.24346700888019	2005	historical
Cairns	24.99	82.55764725404596	2006	historical
Cairns	25.03	82.94573743852062	2007	historical
Cairns	25.25	83.54427222126715	2008	historical
Cairns	25.23	82.85839815345739	2009	historical
Cairns	26.04	82.8477227097823	2010	historical
Cairns	24.84	82.82069101821834	2011	historical
Cairns	25.02	83.06957885696156	2012	historical
Cairns	25.31	83.02072011554239	2013	historical
Cairns	25.06	83.65244731409244	2014	historical
Cairns	25.32	83.2554773037558	2015	historical
Cairns	26.08	82.50282577484428	2016	historical
Cairns	25.72	82.1622612950693	2017	historical
Cairns	25.41	82.46337913083217	2018	historical
Cairns	25.22	82.29079688336616	2019	historical

Table 23: **2030s: Biological Threshold 31.5°C & Fitness Cost 0%**

Location	Temperature	RES	Year	Scenario
Cairns	25.79	82.2776490504004	2024	RCP4.5
Cairns	25.87	82.27755863037292	2024	RCP8.5
Cairns	25.65	82.92913149107211	2025	RCP8.5
Cairns	25.58	82.96063075781612	2025	RCP4.5
Cairns	25.94	82.4994150901891	2026	RCP4.5
Cairns	26.02	82.24329684284014	2026	RCP8.5
Cairns	25.62	82.93400289242966	2027	RCP8.5
Cairns	25.55	83.00368742423463	2027	RCP4.5
Cairns	25.56	83.5432809672251	2028	RCP4.5
Cairns	25.63	83.56565539116204	2028	RCP8.5
Cairns	26.18	82.61837933290167	2029	RCP8.5
Cairns	26.1	82.6183664560521	2029	RCP4.5
Cairns	25.84	82.43444138777281	2030	RCP4.5
Cairns	25.91	82.44204682216524	2030	RCP8.5
Cairns	25.14	82.98331057940855	2031	RCP8.5
Cairns	25.06	83.04902416207936	2031	RCP4.5
Cairns	26.57	82.64308782254128	2032	RCP4.5
Cairns	26.64	82.64369630654484	2032	RCP8.5
Cairns	25.55	82.78562764363609	2033	RCP4.5
Cairns	25.63	82.75008685188592	2033	RCP8.5
Cairns	25.44	82.43538592651373	2034	RCP4.5
Cairns	25.51	82.37479244841626	2034	RCP8.5
Cairns	25.82	82.87481941645703	2035	RCP4.5
Cairns	25.89	82.80447416634966	2035	RCP8.5
Cairns	26.02	82.38206382353455	2036	RCP8.5
Cairns	25.95	82.35394671291454	2036	RCP4.5
Cairns	25.97	82.6322052138121	2037	RCP4.5
Cairns	26.04	82.6043033609292	2037	RCP8.5
Cairns	25.8	82.9486809498261	2038	RCP8.5
Cairns	25.73	82.96910185049015	2038	RCP4.5
Cairns	26.2	82.69673415707935	2039	RCP8.5
Cairns	26.13	82.7009828605501	2039	RCP4.5

Table 24: **2050s: Biological Threshold 31.5°C & Fitness Cost 0%**

Location	Temperature	RES	Year	Scenario
Cairns	26.2	82.42012832029275	2044	RCP4.5
Cairns	26.55	82.78037730832855	2044	RCP8.5
Cairns	26.33	82.49723745944151	2045	RCP8.5
Cairns	25.98	82.72449989029597	2045	RCP4.5
Cairns	26.7	82.67846996221878	2046	RCP8.5
Cairns	26.35	82.35054254171719	2046	RCP4.5
Cairns	26.3	81.69466312878649	2047	RCP8.5
Cairns	25.95	82.47199814462772	2047	RCP4.5
Cairns	26.31	83.62259236998257	2048	RCP8.5
Cairns	25.96	83.72276957541938	2048	RCP4.5
Cairns	26.86	82.62288156231565	2049	RCP8.5
Cairns	26.51	82.74357296448858	2049	RCP4.5
Cairns	26.24	82.62221809146625	2050	RCP4.5
Cairns	26.59	83.02458116763316	2050	RCP8.5
Cairns	25.47	82.82651418742996	2051	RCP4.5
Cairns	25.82	82.70184801058365	2051	RCP8.5
Cairns	27.33	82.66223326058856	2052	RCP8.5
Cairns	26.98	82.75320958330086	2052	RCP4.5
Cairns	25.96	82.50505929833501	2053	RCP4.5
Cairns	26.31	82.435516941004	2053	RCP8.5
Cairns	25.85	82.30717379607124	2054	RCP4.5
Cairns	26.2	82.33044561511788	2054	RCP8.5
Cairns	26.57	82.40590439520406	2055	RCP8.5
Cairns	26.22	82.60368331379482	2055	RCP4.5
Cairns	26.71	82.6756162041182	2056	RCP8.5
Cairns	26.36	82.38734847624467	2056	RCP4.5
Cairns	26.72	82.78488439327026	2057	RCP8.5
Cairns	26.37	82.61289976171491	2057	RCP4.5
Cairns	26.14	83.00395008324199	2058	RCP4.5
Cairns	26.49	83.19989477708603	2058	RCP8.5
Cairns	26.54	82.76671621617572	2059	RCP4.5
Cairns	26.89	82.79474677539277	2059	RCP8.5

Table 25: **Historical: Biological Threshold 31.5°C & Fitness Cost 20%**

Location	Temperature	RES	Year	Scenario
Cairns	25.08	79.6964778412146	1990	historical
Cairns	24.86	80.67595193068017	1991	historical
Cairns	25.23	79.91383695747116	1992	historical
Cairns	24.83	81.44492817299772	1993	historical
Cairns	24.84	80.77484788135465	1994	historical
Cairns	25.39	79.94949244096851	1995	historical
Cairns	25.12	79.64351040816591	1996	historical
Cairns	24.35	81.31423912492033	1997	historical
Cairns	25.85	80.20263001738579	1998	historical
Cairns	24.84	80.58914450558571	1999	historical
Cairns	24.72	80.42872030440174	2000	historical
Cairns	25.1	81.32599407061139	2001	historical
Cairns	25.23	79.88937970252486	2002	historical
Cairns	25.25	80.32849597316681	2003	historical
Cairns	25.01	80.36741719589581	2004	historical
Cairns	25.41	80.42744465461558	2005	historical
Cairns	24.99	79.57018787650051	2006	historical
Cairns	25.03	79.98469581057907	2007	historical
Cairns	25.25	80.74749849067321	2008	historical
Cairns	25.23	79.96152725744776	2009	historical
Cairns	26.04	79.96018772070266	2010	historical
Cairns	24.84	79.81881820561273	2011	historical
Cairns	25.02	80.11498939569645	2012	historical
Cairns	25.31	80.12641518638208	2013	historical
Cairns	25.06	80.94472463314628	2014	historical
Cairns	25.32	80.4435805860594	2015	historical
Cairns	26.08	79.50751158136318	2016	historical
Cairns	25.72	79.00912794120133	2017	historical
Cairns	25.41	79.52683539949882	2018	historical
Cairns	25.22	79.21106275298189	2019	historical

Table 26: **2030s: Biological Threshold 31.5°C & Fitness Cost 20%**

Location	Temperature	RES	Year	Scenario
Cairns	25.87	79.13078384296543	2024	RCP8.5
Cairns	25.79	79.13726210200701	2024	RCP4.5
Cairns	25.65	80.05688206316222	2025	RCP8.5
Cairns	25.58	80.09480315637661	2025	RCP4.5
Cairns	25.94	79.3803413439068	2026	RCP4.5
Cairns	26.02	79.07654863395445	2026	RCP8.5
Cairns	25.55	80.16357048557316	2027	RCP4.5
Cairns	25.62	80.07063121594612	2027	RCP8.5
Cairns	25.63	80.77555361558716	2028	RCP8.5
Cairns	25.56	80.75783009517656	2028	RCP4.5
Cairns	26.18	79.60187292679333	2029	RCP8.5
Cairns	26.1	79.60911156126984	2029	RCP4.5
Cairns	25.91	79.40419516729199	2030	RCP8.5
Cairns	25.84	79.39811382010545	2030	RCP4.5
Cairns	25.06	80.16653269130803	2031	RCP4.5
Cairns	25.14	80.08413421896002	2031	RCP8.5
Cairns	26.57	79.65063472060153	2032	RCP4.5
Cairns	26.64	79.63920057198452	2032	RCP8.5
Cairns	25.63	79.77860708420216	2033	RCP8.5
Cairns	25.55	79.82618883037887	2033	RCP4.5
Cairns	25.51	79.29611607228058	2034	RCP8.5
Cairns	25.44	79.37138252223203	2034	RCP4.5
Cairns	25.89	79.893956893995	2035	RCP8.5
Cairns	25.82	79.98285531041365	2035	RCP4.5
Cairns	25.95	79.20175314426507	2036	RCP4.5
Cairns	26.02	79.23236117657619	2036	RCP8.5
Cairns	25.97	79.6705666716735	2037	RCP4.5
Cairns	26.04	79.63431252312223	2037	RCP8.5
Cairns	25.73	80.03750511521255	2038	RCP4.5
Cairns	25.8	80.00388640998186	2038	RCP8.5
Cairns	26.13	79.74042671554295	2039	RCP4.5
Cairns	26.2	79.72652317548082	2039	RCP8.5

Table 27: **2050s: Biological Threshold 31.5°C & Fitness Cost 20%**

Location	Temperature	RES	Year	Scenario
Cairns	26.2	82.42012832029275	2044	RCP4.5
Cairns	26.55	82.78037730832855	2044	RCP8.5
Cairns	26.33	82.49723745944151	2045	RCP8.5
Cairns	25.98	82.72449989029597	2045	RCP4.5
Cairns	26.7	82.67846996221878	2046	RCP8.5
Cairns	26.35	82.35054254171719	2046	RCP4.5
Cairns	26.3	81.69466312878649	2047	RCP8.5
Cairns	25.95	82.47199814462772	2047	RCP4.5
Cairns	26.31	83.62259236998257	2048	RCP8.5
Cairns	25.96	83.72276957541938	2048	RCP4.5
Cairns	26.86	82.62288156231565	2049	RCP8.5
Cairns	26.51	82.74357296448858	2049	RCP4.5
Cairns	26.24	82.62221809146625	2050	RCP4.5
Cairns	26.59	83.02458116763316	2050	RCP8.5
Cairns	25.47	82.82651418742996	2051	RCP4.5
Cairns	25.82	82.70184801058365	2051	RCP8.5
Cairns	27.33	82.66223326058856	2052	RCP8.5
Cairns	26.98	82.75320958330086	2052	RCP4.5
Cairns	25.96	82.50505929833501	2053	RCP4.5
Cairns	26.31	82.435516941004	2053	RCP8.5
Cairns	25.85	82.30717379607124	2054	RCP4.5
Cairns	26.2	82.33044561511788	2054	RCP8.5
Cairns	26.57	82.40590439520406	2055	RCP8.5
Cairns	26.22	82.60368331379482	2055	RCP4.5
Cairns	26.71	82.6756162041182	2056	RCP8.5
Cairns	26.36	82.38734847624467	2056	RCP4.5
Cairns	26.72	82.78488439327026	2057	RCP8.5
Cairns	26.37	82.61289976171491	2057	RCP4.5
Cairns	26.14	83.00395008324199	2058	RCP4.5
Cairns	26.49	83.19989477708603	2058	RCP8.5
Cairns	26.54	82.76671621617572	2059	RCP4.5
Cairns	26.89	82.79474677539277	2059	RCP8.5

References

1. Ulrich, J. N., Beier, J. C., Devine, G. J. & Hugo, L. E. Heat sensitivity of w Mel Wolbachia during Aedes aegypti development. *PLoS neglected tropical diseases* **10**, e0004873 (2016).
2. Nairne, J. & Fawcett, R. *Defining heatwaves: heatwave defined as a heat-impact event servicing all community and business sectors in Australia. The Centre for Australia Weather and Climate Research*. (2013).
3. The World Bank Group. Climate Change Knowledge Portal. (2021).
4. Rossi, M. M., Ólivêr, L. & Massad, E. Modelling the implications of temperature on the life cycle of Aedes aegypti mosquitoes. in *Ecological Modelling Applied to Entomology* 81–107 (Springer, 2014).
5. Sánchez C., H. M. Mosquito Gene Drive Explorer. (2020).
6. Sánchez C., H. M., Wu, S. L., Bennett, J. B. & Marshall, J. M. MGDrivE: A modular simulation framework for the spread of gene drives through spatially explicit mosquito populations. *Methods in Ecology and Evolution* **11**, 229–239 (2020).
